# Interspecies Differences in Proteome Turnover Kinetics Are Correlated With Life Spans and Energetic Demands

**DOI:** 10.1074/mcp.RA120.002301

**Published:** 2021-01-07

**Authors:** Kyle Swovick, Denis Firsanov, Kevin A. Welle, Jennifer R. Hryhorenko, John P. Wise, Craig George, Todd L. Sformo, Andrei Seluanov, Vera Gorbunova, Sina Ghaemmaghami

**Affiliations:** 1 Department of Biology, University of Rochester, Rochester, New York, USA; 2 Mass Spectrometry Resource Laboratory, University of Rochester, Rochester, New York, USA; 3 Department of Pharmacology and Toxicology, Wise Laboratory for Environmental and Genetic Toxicology, University of Louisville, Louisville, Kentucky, USA; 4 North Slope Borough Department of Wildlife Management, Barrow, Alaska, USA; 5 Institute of Arctic Biology, University of Alaska Fairbanks, Fairbanks, Alaska, USA

**Keywords:** Protein turnove, aging, protein degradation, proteostasis, quantitative proteomics, ACN, acetonitrile, AF, ammonium formate, AGC, automatic gain control, AZC, azetidine-2-carboxylic acid, DMEM, Dulbecco’s modified Eagle’s medium, EMEM, Eagle’s minimum essential medium, ETC, electron transport chain, FBS, fetal bovine serum, GO, gene ontology, H/L, heavy-to-light, iBAQ, intensity-based absolute quantification, LFQ, label-free quantification, OCR, oxygen consumption rate, PER, proton efflux rate, ROS, reactive oxygen species, SILAC, stable isotopic labeling in cell culture, TLS, turnover–life span slope, UPS, ubiquitin–proteasome system

## Abstract

Cells continually degrade and replace damaged proteins. However, the high energetic demand of protein turnover generates reactive oxygen species that compromise the long-term health of the proteome. Thus, the relationship between aging, protein turnover, and energetic demand remains unclear. Here, we used a proteomic approach to measure rates of protein turnover within primary fibroblasts isolated from a number of species with diverse life spans including the longest-lived mammal, the bowhead whale. We show that organismal life span is negatively correlated with turnover rates of highly abundant proteins. In comparison with mice, cells from long-lived naked mole rats have slower rates of protein turnover, lower levels of ATP production, and reduced reactive oxygen species levels. Despite having slower rates of protein turnover, naked mole rat cells tolerate protein misfolding stress more effectively than mouse cells. We suggest that in lieu of a rapid constitutive turnover, long-lived species may have evolved more energetically efficient mechanisms for selective detection and clearance of damaged proteins.

## Highlights

• Cross-species proteomic analyses show that turnover is correlated with life span.
• Correlation between life span and turnover is evident for abundant proteins.
• Long-lived naked mole rats also have lower ATP production and ROS levels.
• Despite having slow turnover, naked mole rats tolerate protein misfolding stress.

## Graphical abstract

**Figure:**
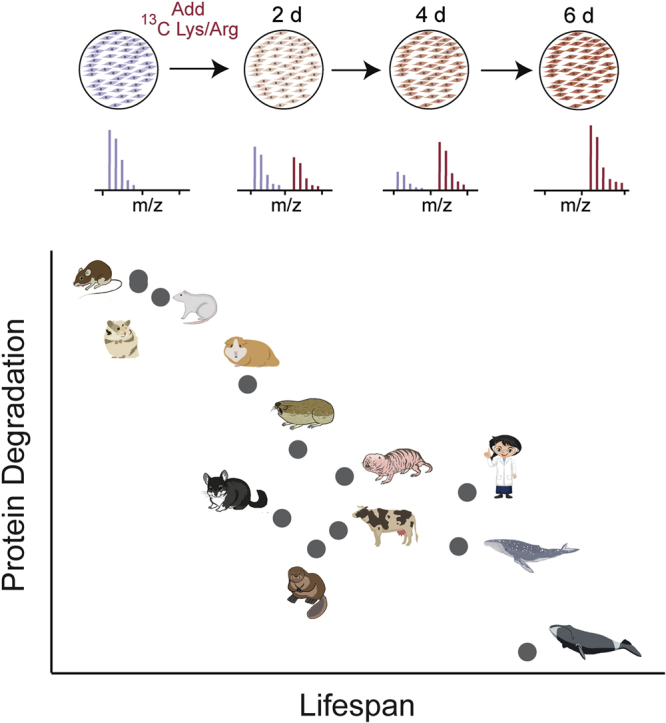

## In Brief

A proteomic approach was used to measure protein turnover rates within fibroblasts isolated from species with diverse life spans. We found that life span is negatively correlated with turnover rates of highly abundant proteins. Despite having slower rates of protein turnover, long-lived naked mole rat cells tolerate protein misfolding stress more effectively than short-lived mouse cells. We suggest that in lieu of rapid constitutive turnover, long-lived species have evolved more energetically efficient mechanisms for selective detection and clearance of damaged proteins.

Protein homeostasis (proteostasis) encompasses an array of coordinated cellular functions that ensure the proper synthesis, folding, and degradation of cellular proteins (1, 2). These functions are carried out by a number of cellular pathways that include the ribosomal translational machinery, the cellular chaperone network, the ubiquitin–proteasome system (UPS) and autophagic degradation (3). Combined, these pathways are required to maintain a functional proteome throughout the life span of an organism. A key feature of proteostasis is protein turnover, the process by which cellular proteins are continuously degraded and replaced by newly synthesized proteins (4). Because of its central role in protein quality control, there has been significant interest in understanding the relationship between alterations in protein turnover kinetics and proteostatic disruptions that occur during aging (1, 5, 6, 7).

Within a cell, proteins have widely varying half-lives ranging from minutes to years (8, 9, 10). Protein half-lives are established by a complex combination of intrinsic and extrinsic factors including degradative sequence motifs, the folding state of the protein, and activities of cellular proteolytic pathways (11, 12, 13, 14, 15, 16, 17). Protein half-lives are not only divergent within a proteome but also can vary for the same protein expressed in different organisms, tissues, cell types, and environmental conditions (9, 18, 19, 20, 21). It is thought that the constitutive clearance and replacement of cellular proteins accounts for the majority of their degradative flux within a cell (22, 23). In addition to this basal turnover, cells maintain a number of sophisticated pathways for specific detection and selective clearance of proteins that have been damaged through covalent modifications or detrimental conformational alterations (3, 24, 25). Together, the constitutive and selective clearance of proteins are important for maintaining a healthy proteome within a cell, and alterations in protein turnover have been associated with the accumulation of damaged proteins and their pathogenic aggregation during aging and age-associated diseases (5, 6, 26, 27, 28).

Quantitative analyses of protein turnover have been significantly bolstered by recent technical advances in mass spectrometry–based proteomics. The use of stable isotope labeling in conjunction with LC-MS/MS has opened the door for analyses of protein turnover kinetics on a global scale (4, 9, 17, 18, 29). In a number of model systems, proteomic studies have shown that aged individuals have a significantly slower protein turnover than younger counterparts (30, 31, 32), although this effect may not be universal and appears variable for different proteins, tissues, and organisms (33, 34, 35, 36). More mechanistic studies have linked the age-dependent decrease in protein turnover and resulting loss in proteostasis to alterations in specific degradation pathways including the UPS and autophagy (35, 37, 38).

The above studies of naturally aging organisms suggest that slower rates of protein turnover may be associated with less robust protein quality control and diminished proteostasis during aging. This trend is echoed in studies of invertebrates whose life spans have been artificially prolonged by genetic alterations or life-extending treatments. For example, the ablation of the insulin-like growth factor 1 receptor *daf2* in *Caenorhabditis elegans* extends life span and results in an increase in global protein turnover rates, counteracting the natural decline in the turnover that occurs during aging (31, 39, 40, 41). In apparent contrast, a number of studies have shown that protein turnover rates are actually reduced in mammalian model systems whose life spans have been extended by dietary regimens, therapeutic interventions, and genetic modifications (5). For example, mice that have been treated with rapamycin or calorie restriction, both well-documented methods of life span extension, have reduced protein turnover rates (42, 43, 44, 45). Similarly, in long-lived Snell dwarf mutant (*Pit1*^*dw*^) mice, rates of protein turnover are diminished (45). Thus, the nature of the relationship between protein turnover and longevity appears complex and remains enigmatic.

Within mammals, why are faster rates of protein turnover generally associated with youth, but organisms that are able to live longer have slower rates of protein turnover? To gain insight into this question, our laboratory previously conducted a comparative proteomic study of fibroblasts cultured from a number of rodent species with diverse life spans and showed that within this limited set of organisms, longevity was correlated with slower rates of *in vitro* protein turnover (21). However, the generality and underlying basis of this trend were unclear. In the present study, we have expanded our cross-species comparisons of protein turnover kinetics to include mammalian species outside the order Rodentia, including the longest-lived mammal, the bowhead whale (*Balaena mysticetus*). We show that the negative correlation between life span and protein turnover rates is apparent in mammalian species with life spans ranging from 3 to 200 years. By comparing a representative pair of short-lived and long-lived species (mouse and naked mole rat, respectively), we investigate the ramifications of slower protein turnover on cellular ATP demand, generation of reactive oxygen species (ROS), and the ability to survive protein misfolding stress. The results provide insights into the proteostatic costs and benefits of protein turnover and its relationship to longevity in mammals.

## Experimental Procedures

### Experimental Design and Statistical Rationale

In this study, we conducted proteomic experiments to compare protein degradation rates, steady-state protein levels, and incorporation of the proteotoxic proline analogue azetidine-2-carboxylic acid (AZC) within a number of mammalian species (supplemental Fig. S1). The theoretical rationales and assay workflows for these proteomic experiments are discussed in individual sections in Experimental Procedures and Results sections. Briefly, for analyses of protein degradation, cultures of dermal fibroblasts isolated from each species were grown to confluency and labeled for varying times (0 days, 2 days, 4 days, and 6 days) in labeling media. The fractional labeling of the peptides was analyzed at the MS1 level using the procedures described below (also see (21)). The determination of the rate constants for fractional labeling were conducted by least squares regression analysis of the time-resolved data fitted to equations derived from the kinetic model described previously (21), and the goodness of fit was assessed by R^2^ values. To examine the reproducibility of rate measurements within individuals of a given species, dermal fibroblasts from two different bowhead whales were isolated and analyzed. Correlations of rate constants between species were measured using Spearman rank correlation coefficients as measured degradation rates were typically not normally distributed. To determine the relationship between organismal life span and turnover rates of individual proteins, least squares regression analyses were performed as described below. Proteomic analyses of protein abundances were conducted using intensity-based absolute quantification (iBAQ) using protein standards with known concentrations (Universal Proteomics Standard 2, Sigma-Aldrich, see below) as previously described (46). Incorporation of AZC into proteins was analyzed by searching for variable proline modifications with the predicted mass change as described below. To examine the false discovery rate for this modification, cells treated with ^13^C-proline and DMSO were analyzed as controls. The number of technical and biological replicates for low-throughput cell-based and biochemical assays examining metabolic activity, cell viability, protein expression levels, ROS levels, and ubiquitination levels is stated in the corresponding sections below. The statistical significance (*p* values) of variations in these measurements was analyzed by Mann–Whitney *U* tests unless otherwise stated.

### Cell Culture

Dermal fibroblasts were isolated and cultured according to previously described protocols (21, 47). Briefly, tissues were shaved and cleaned with 70% ethanol and then washed with Dulbecco’s modified Eagle’s medium (DMEM)/F-12 medium (Thermo Fisher) with Liberase (Sigma) at 37 °C on a stirrer for 15 to 90 min. Tissues were then washed and plated with DMEM/F-12 medium containing 15% fetal bovine serum (FBS) (GIBCO) and Antibiotic-Antimycotic (GIBCO). Within two passages, they were frozen in liquid nitrogen when they reached ∼80% confluency. All cultures were propagated and expanded in Eagle’s minimum essential medium (EMEM) supplemented with 15% FBS, 100 U/ml penicillin, and 100 U/ml streptomycin at 37 °C with 5% CO_2_ and 3% O_2_ except naked mole rat and whale cells, which were initially propagated at 32 °C with 5% CO_2_ and 3% O_2_ before being acclimated to 37 °C for 4 days. All subsequent experiments were carried out at 37 °C for all species.

### Isotopic Labeling

Before isotopic labeling, cultures were grown to confluency. Once cells ceased cell division and were contact inhibited, they were maintained in a quiescent state for 4 days. Subsequently, cells were acclimated to the labeling media (EMEM) supplemented with 15% dialyzed FBS (Thermo Scientific), 100 U/ml penicillin, and 100 U/ml streptomycin for 4 days. After 4 additional days in the adaptation media, cultures were introduced to the minimum essential medium for stable isotopic labeling in cell culture (SILAC) (Thermo Scientific) supplemented with L-arginine:HCl (^13^C6, 99%) and L-lysine:2HCl (^13^C6, 99%; Cambridge Isotope Laboratories) at concentrations of 0.13 g/l and 0.0904 g/l, respectively, 15% dialyzed FBS (Thermo Scientific), 100 U/ml penicillin, and 100 U/ml streptomycin. After 0, 2, 4, and 6 days of labeling, cells were harvested and washed with PBS, and cell pellets were frozen before further analysis.

### Mass Spectrometry Sample Preparation

Cells were lysed in 8 M urea, 150-mM NaCl, and 50-mM Hepes (pH = 9.0). Cell pellets were resuspended in 50 μl of the lysis buffer per 10^6^ cells and sonicated three times using a high-energy sonicator (QSonica, Newtown, CT) for 10 s with 60-s resting periods on ice. The extracts were centrifuged for 5 min at 16,000*g*, and supernatants were transferred to new Eppendorf tubes. Protein concentrations were measured using a bicinchoninic assay kit (Thermo Scientific). Subsequent experiments were performed using 50 μg of total protein from each extract. Disulfide bonds were reduced with 5-mM Tris(2-carboxyethyl)phosphine Bond-Breaker (Thermo Scientific) at RT for 1 h, and proteins were alkylated with 10-mM iodoacetamide at RT for 30 min in darkness. DTT (1 mM) was added to quench iodoacetamide, and samples were diluted to a urea concentration of less than 1 M with 50-mM Hepes. To derive tryptic peptides, 1 μg of trypsin (selective cleavage on the C-terminal side of lysine and arginine residues) was added to samples before overnight incubation at 37 °C. To quench trypsin, formic acid was added to a final concentration of 1%.

To increase proteome coverage, high-pH fractionation was conducted on extracts before LC-MS/MS using homemade C18 spin columns. Eight different elution buffers were made with 100-mM ammonium formate (pH 10) (AF) with 5%, 7.5%, 10%, 12.5%, 15%, 17.5%, 20%, and 50% acetonitrile (ACN) added. After conditioning the column with ACN and AF, samples were added and centrifuged. An AF wash was performed to remove any residual salt before the eight elutions were collected in fresh tubes. All fractions were then dried and resuspended in 10 μl of 0.1% TFA. The LC-MS/MS method used for each type of experiment is described at the end of the Experimental Procedures section.

### Data Analysis of Isotopic Labeling Kinetics

MS2 data for all samples were searched against the appropriate UniProt database using the integrated Andromeda search engine with MaxQuant software, version 1.5.8.3 (48). For all species, searches were conducted against both the *Mus musculus* database (22,305 entries, downloaded 8/7/2017) as well as species-specific databases. For species where well-annotated sequence databases were not available, the latter searches were conducted against the closest related species. For example, the bowhead whale, humpback whale, and cow data were searched against the *Bos taurus* database (20,612 entries, downloaded 4/8/2019), whereas the human data were searched against the *Homo sapiens* database (23,848 entries, downloaded 3/15/2020). All cross-species comparative analyses presented in this article use the search results against the *M. musculus* database. Although this approach reduces the coverage of proteomic data for non–mouse species, it avoids potential errors in false assignment of protein orthology when conducting interspecies comparisons of protein *k*_*deg*_ measurements. Our previous analyses of rodent species had indicated that the distribution and median of *k*_*deg*_ measurements for a given species were similar regardless of whether searches were conducted against species-specific or mouse databases (21). The results of all searches against mouse and species-specific databases are available on the Proteomics Identification database (accession number PXD018325).

The maximum allowable numbers of missed cleavages in all searches were two and the false discovery rate thresholds were set to maximum of 1%. SILAC peptide and protein quantifications were performed with MaxQuant using default parameters (available on the Proteomics Identification database). For each peptide, heavy-to-light (H/L) SILAC ratio was determined by MaxQuant for all peptides where both H and L intensities were greater than zero. The H/L ratios obtained by MaxQuant were subsequently converted to fraction-labeled (H/H + L) measurements.

The determination of degradation rate constants (*k*_*deg*_) from the fraction-labeled measurements was conducted in accordance to the kinetic model outlined previously (9, 17, 21). To obtain *k*_*deg*_ measurements for each peptide, plots of the fraction labeled as a function of time were fitted to a single-exponential function ($fraction\;labeled\;\left(t\right)=1-\;e^{-k_{deg}*t}$) using least squares fitting. To determine *k*_*deg*_ at the protein level, the median fraction-labeled measurements of all peptides mapped to specific proteins were fitted to the above function. All reported *k*_*deg*_ measurements at the protein level were required to pass three quality control criteria:

1. A nonzero H/L ratio was calculated in at least two unique peptide sequences;
2. The fraction labeled was quantified in two or more time points in addition to the unlabeled time point;
3. The R^2^ of the fitted function was greater than 0.80.

Turnover–life span slopes (TLSs) were measured as described in the Results section, and gene ontology (GO) enrichment analyses were conducted on genes ranked based on their TLS values using GOrilla (49).

### Measuring Steady-State Protein Levels

Three separate cultures were grown to quiescence in the EMEM supplemented with 15% FBS, 100 U/ml penicillin, and 100 U/ml streptomycin. Cell extracts were prepared for LC-MS/MS analysis as described above. The LC-MS/MS method is described at the end of the Experimental Procedures section. Data were searched against the *M. musculus* UniProt database, and peptide and protein quantifications were performed with MaxQuant. All identified peptides that shared 100% sequence homology between mouse and naked mole rat were then used in subsequent analyses. Log_2_ fold change and *p*-values were calculated using the Proteus software (50) using the default settings and normalized by quantile normalization. GO enrichment analyses were conducted on genes ranked based on their Log_2_ fold change values using GOrilla (49).

To measure absolute protein levels, fibroblasts were grown to quiescence at 37 °C in the EMEM supplemented with 15% FBS, 100 U/ml penicillin, and 100 U/ml streptomycin. The Universal Proteomics Standard 2 (Sigma-Aldrich) was dissolved in the lysis buffer (8-M urea and 150-mM and 50-mM Hepes (pH = 9.0) and 4.24 μg was added to 13 μg of lysate. The resulting mixture was prepared and analyzed as described above. MaxQuant protein intensities were divided by the number of theoretically observable peptides. The resulting iBAQ intensities of the standards were then log-transformed and plotted against the known log-transformed molar values of the standards. Linear regression was used to fit iBAQ intensities of the standards to the known molar protein amounts. From the resulting curve, the slope and y-intercept were used to convert iBAQ values to molar amounts for identified proteins.

### Western Blots

20 μg of each extract was analyzed by electrophoresis in 10% polyacrylamide gels and transferred to polyvinylidene difluoride membranes using the Trans-Blot SD Semi-Dry Electrophoretic Transfer Cell (Bio Rad). After 1-h incubation at RT in Odyssey Blocking Buffer (TBS) (Li-Cor Biosciences), the membrane was incubated with the indicated antibodies at 4 °C overnight. The membranes were then washed with Tris-bufferd saline, 0.1% Tween 20, and the corresponding secondary antibodies were applied to the membranes for 1 h at RT. The membranes were then washed with Tris-bufferd saline, 0.1% Tween 20, and the detection of signals was performed using Odyssey Scanning Instrument (Li-Cor Biosciences). The primary antibodies and their corresponding dilutions were as follows. Anti-alpha Tubulin antibody: 1:1000 (ab4074, Abcam), Recombinant Anti-ENO1 antibody [EPR19758]: 1:1000 (ab227978, Abcam), GAPDH Monoclonal Antibody (6C5): 1:1000 (AM4300, Invitrogen), UQCRC1 Monoclonal Antibody (16D10AD9AH5): 1:1000 (459140, Invitrogen), Recombinant Anti-ATP5L2 + ATP5L antibody [EPR15636]: 1:1000 (ab191417, Abcam), COX4 Monoclonal Antibody (K.473.4): 1:1000 (MA5-15078, Invitrogen), and Ubiquitin Monoclonal Antibody (P4D1): 1:1000 (3936S, Cell Signaling Technology).

### ATP Production Rates

Two days before measuring ATP production rates, mouse and naked mole rat cells were cultured on a Seahorse XF96 Cell Culture Microplate (Agilent) at densities of 105,000 and 80,150 cells per well, respectively, and grown to confluency. 24 h before measuring ATP production, the media was changed to DMEM (Corning) supplemented with 15% FBS, 100 U/ml penicillin, and 100 U/ml streptomycin. At the same time, an XFe96 Sensor Cartridge was hydrated with sterile water and placed in a non-CO_2_ 37 °C incubator. On the day of measuring ATP production, 1 ml each of 1 M glucose, 100-mM pyruvate, and 200-mM L-glutamine were added to XF DMEM (Agilent). Cells were rinsed and adapted to XF DMEM for 1 h before analysis. The sensor cartridge was incubated in a non-CO_2_ 37 °C incubator for 1 h with the sensors in XF Calibrant. 1.5-μM oligomycin was added to port A and 0.5-μM rotenone/antimycin A was added to port B. After 1 h, the plate and sensor cartridges were loaded onto a Seahorse XFe analyzer and an XF Real-Time ATP Rate Assay was performed according to manufacturer’s specifications. To normalize the oxygen consumption rate (OCR) and proton efflux rate (PER) measurements, total protein amounts per cell were measured by determining cell numbers, cell weights, and protein concentrations in each well. Cell numbers were measured using a Celigo Imaging Cytometer (Nexcelom Bioscience) after staining by Hoechst 33342 (Cell Signaling Technology), and protein concentrations were measured by bicinchoninic assays. A total of 44 individual wells were analyzed for each species. In addition, eight blank wells were analyzed to correct for the background signal.

### Quantifying Cellular ROS

Mouse and naked mole rat cultures were grown to quiescence, treated with 5 μM of CellROX Orange Reagent (Thermo Fisher) and incubated for 30 min at 37 °C. Subsequently, the media was aspirated and cells were washed three times with PBS. Cells were harvested using trypsin and centrifuged at 300*g* for 5 min. Resulting pellets were resuspended in PBS and transferred to new tubes. To quantify fluorescence, the Oxidative Stress program was run on a Muse Cell Analyzer (Luminex). To measure background autofluorescence, cells not treated with CellROX were also analyzed. For cells treated with CellROX, only those with fluorescence levels higher than the background level were analyzed. To ensure cellular debris and dead cells did not contribute to the overall fluorescence measurements, appropriate gates were used to exclude their selection. For analysis of biological replicate 2, confluent cells were treated with CellROX as described above, harvested and resuspended in PBS containing 1 μg/ml 4,6-diamidino-2-phenylindole (DAPI) (Thermo Fisher) for exclusion of dead cells, and were kept in dark on ice until analysis. Flow cytometry analysis was performed on an LSR II instrument (BD Biosciences). At least 20,000 events of similarly sized cells were collected for each sample. FlowJo 7.6 (BD Biosciences) software was used for data analysis.

### Mitochondrial Staining

Mouse and naked mole rat cells were grown to confluency. Complete culture media containing 10-nM MitoTracker Deep Red (Thermo Fisher) was added to cells, and cultures were incubated for 30 min at 37 °C. After incubation, cells were washed twice with PBS, harvested with trypsin, and centrifuged at 300*g* for 5 min. Pellets were resuspended in PBS containing 1 μg/ml DAPI (Thermo Fisher) to exclude dead cells and immediately placed on ice before analysis. Flow cytometry analysis was performed on the LSR II instrument (BD Biosciences). Dead cells and cellular debris were excluded from analysis by using appropriate gating. At least 20,000 events of similarly sized cells were collected for each sample. FlowJo 7.6 (BD Biosciences) software was used for data analysis.

### AZC Treatment and Cell Viability

To measure viability of dividing cells in response to AZC (Sigma-Aldrich), cultures were grown in the presence of varying concentrations of AZC. Two days after plating, cells were harvested and cell numbers were calculated. To measure cell viability, the total number of cells was compared with cells with no AZC added. To measure viability of quiescent cells in response to AZC, cells were treated with varying concentrations of AZC and after 5 days of treatment, viability was measured using trypan blue (Thermo Fisher) exclusion. To measure exclusion, 100 μl of a 0.4% trypan blue solution was added to 100 μl of cells, and the resulting mixture was loaded onto a hemacytometer. To calculate cell viability, the number of blue cells was divided by the total number of cells. For each species, the experiment was conducted for three biological replicates. For each biological replicate, measurements were made for four technical replicates and the average cell viability was calculated.

To measure viability of quiescent cells in response to AZC and MG115 treatment, mouse and naked mole rat cells were cultured as described above and treated with 5 μM of MG115 (Abcam) for 24 h before treating with varying concentrations of AZC. The media was changed every day with fresh MG115 and AZC. After 5 days of AZC treatment, cells were harvested and viability was determined as described above.

### Measuring AZC Incorporation

Eight plates of mouse and naked mole rat fibroblast cultures were grown to quiescence as described above. Four days after shifting quiescent naked mole rat cultures to 37 °C, four plates of each species were treated with 2.5-mM L-proline (^13^C5, 99%; Cambridge Isotope Laboratories) and four plates were treated with 2.5-mM AZC. After 3, 4, 8, and 16 days of labeling, cells were harvested and washed with PBS, and pellets were frozen before further analysis. Extracts were prepared for and analyzed by LC-MS/MS as described above. All samples were searched against the *M. musculus* UniProt database. To calculate the incorporation of ^13^C5 proline, the fraction of peptides with labeled prolines was calculated as described above for labeled lysines and arginines. To measure AZC incorporation, a proline-specific modification with a mass change of −14.01565 was created within MaxQuant. To obtain a relative metric for the incorporation of AZC within a given tryptic peptide, the intensity of AZC-containing peptides was divided by the intensities of the unmodified versions of the same peptide. To compare global shifts in AZC levels over time, all peptides for which incorporation levels could be calculated in individual time points were used. For pairwise comparisons and Log_2_ ratio calculations, AZC incorporation of peptides with identical sequences was compared.

### Quantitation of Ubiquitinated Proteins in Response to AZC

To measure changes in protein ubiquitination, mouse and naked mole rat cells were grown to quiescence as described above. Cells were either untreated or treated with 2.5-mM AZC for 5 days. Cells were harvested and analyzed by Western blots as described above. Quantification was performed using Image Studio (Li-Cor Biosciences).

To measure changes in protein ubiquitination under proteasomal inhibition, mouse and naked mole rat cells were grown to quiescence as described above. Cells were either treated with DMSO or 5-μM MG115 for 24 h and subsequently treated with 2.5-mM AZC for 5 additional days. Cells were harvested and analyzed by Western blots as described above.

### LC-MS/MS Methods

To measure *k*_*deg*_ of the human, cow, humpback whale, and bowhead whale and label-free quantification (LFQ) of the mouse and naked mole rat, peptides were injected onto a homemade 30-cm C18 column with 1.8-μm beads (Sepax), with an Easy nLC-1200 HPLC (Thermo Fisher), connected to an Orbitrap Fusion Lumos mass spectrometer (Thermo Fisher). Solvent A was 0.1% formic acid in water, whereas solvent B was 0.1% formic acid in 80% ACN. Ions were introduced to the mass spectrometer using a Nanospray Flex source operating at 2 kV. Peptides were eluted off the column using a multistep gradient that began at 3% B and held for 2 min, quickly ramped to 10% B over 5 min, increased to 38% B over 68 min, then ramped to 90% B in 3 min, and was held there for an additional 3 min to wash the column. The gradient then returned to starting conditions in 2 min, and the column was re-equilibrated for 7 min, for a total run time of 90 min. The flow rate was 300 nl/min throughout the run. The Fusion Lumos was operated in a data-dependent mode with a cycle time of 3 s. The full scan was performed over a range of 375 to 1400 m/z, with a resolution of 120,000 at m/z of 200, an automatic gain control (AGC) target of 4e5, and a maximum injection time of 50 ms. Peptides with a charge state between 2 and 5 were selected for fragmentation. Precursor ions were fragmented by collision-induced dissociation using a collision energy of 30 and an isolation width of 1.1 m/z. MS2 scans were collected in the ion trap with the scan rate set to rapid, a maximum injection time of 35 ms, and an AGC setting of 1e4. Dynamic exclusion was set to 20 s to allow the mass spectrometer to fragment lower abundant peptides.

Measuring the incorporation of ^13^C-proline and AZC in mouse and naked mole rat cells was performed as above, with the following minor changes. The gradient was lengthened so that the total run time was 120 min, and the cycle time was reduced to 2 s. Dynamic exclusion was also increased to 45 s.

To perform iBAQ measurements, peptides were injected onto the column described above, with an Easy nLC-1000 HPLC (Thermo Fisher), connected to a Q Exactive Plus mass spectrometer (Thermo Fisher) using the Nanospray Flex source operating at 2 kV. Solvent A was 0.1% formic acid in water, whereas solvent B was 0.1% formic acid in ACN using a flow rate of 300 nl/min. The gradient began at 3% B and held for 2 min, ramped to 8% B over 5 min, increased to 30% B over 68 min, ramped to 70% B in 3 min, and held for 3 min before returning to starting conditions in 2 min. The column was re-equilibrated for 7 min, for a total run time of 90 min. The Q Exactive Plus was operated in data-dependent mode with a full MS scan followed by 20 MS/MS scans. The full scan was performed over a range of 400 to 1400 m/z, with a resolution of 70,000 at m/z of 200, an AGC target of 1e6, and a maximum injection time of 50 ms. Once again, peptides with a charge state between 2 and 5 were selected for fragmentation. Precursor ions were fragmented by higher-energy collisional dissociation (HCD) using a collision energy of 27 and an isolation width of 1.5 m/z, with a 0.3 m/z isolation offset. MS2 were acquired with a resolution of 17,500 at 200 m/z, a maximum injection time of 55 ms, and an AGC setting of 5e4. Dynamic exclusion was set to 25 s.

## Results

### Proteome-Wide Analysis of Protein Degradation Rates (*k*_*deg*_)

**Fig. 1:**
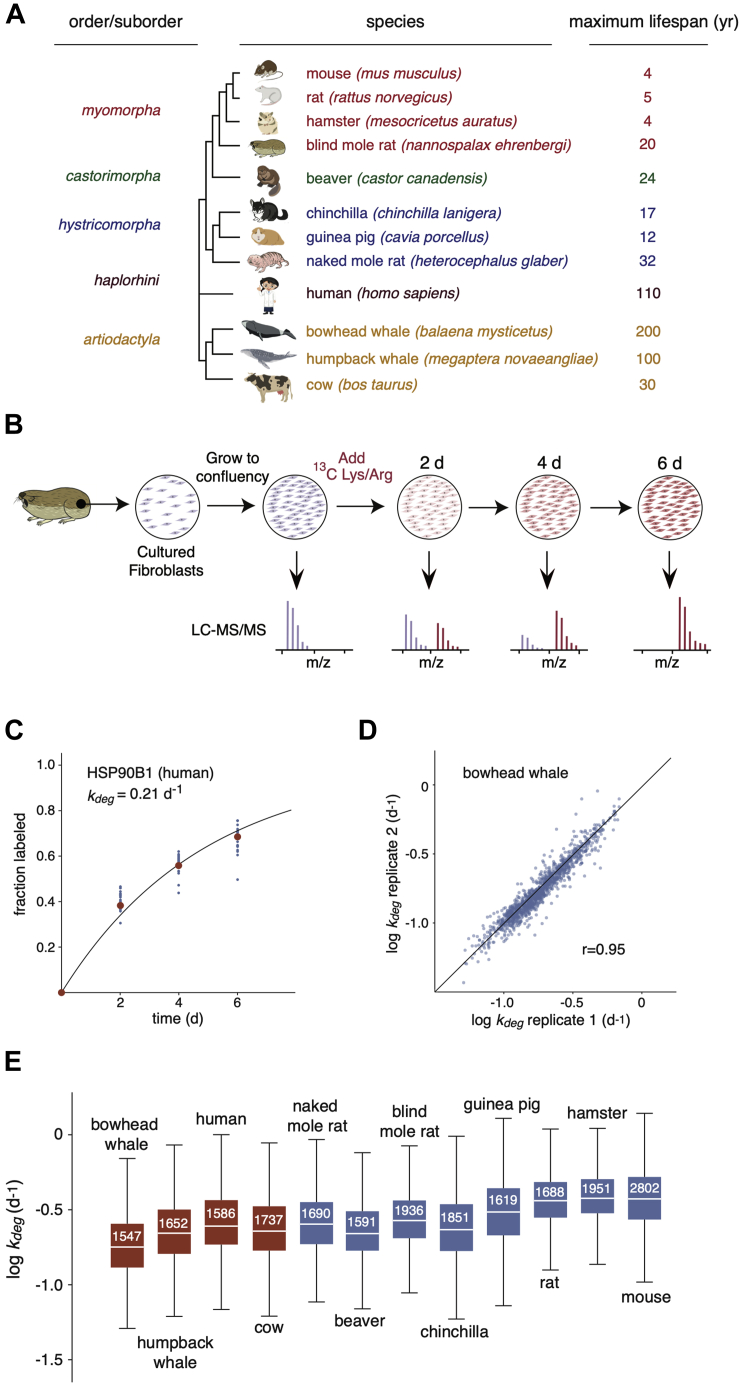
**Proteome-wide quantitation of *k***_***deg***_**in mammalian fibroblasts.***A*, phylogenetic tree and maximal life spans of species analyzed in this study. *Colors* indicate distinct orders and suborders. *B*, dynamic SILAC experimental design. *Blue* and *red* colors indicate unlabeled and isotopically labeled spectra/cells, respectively. *C*, labeling kinetics of human HSP90B1 (endoplasmin) shown as an example of protein-level determination of *k*_*deg*_ values. *Blue dots* indicate the fraction labeled for all peptides mapped to the protein, and *red dots* indicate the median fraction labeled for all peptides. The *blue curve* is a least squares fit to a first-order exponential equation used to measure the indicated *k*_*deg*_ value. *D*, pairwise comparison of *k*_*deg*_ measurements in biological replicates of bowhead whale cells. The *solid line* indicates the identity line. The r value indicates the Pearson correlation coefficient. *E*, distribution of *k*_*deg*_ measurements for each species. The *box plots* indicate the median (*white line*), the interquartile range (*box*), and complete range (*whiskers*) of *k*_*deg*_ measurements excluding far outliers (>2 SD). The number in each box indicates the number of protein-level *k*_*deg*_ measurements. *Red* and *blue boxes* distinguish data collected in this study and the previous study by Swovick *et al.* (21), respectively. SILAC, stable isotopic labeling in cell culture.

Skin fibroblasts were isolated from twelve different mammalian species and propagated in culture as previously described (Fig. 1*A*) (21, 47). The collection included eight rodent species whose proteome-wide turnover kinetics were analyzed in a previous study (21). In addition, the present study included four mammalian species: human (*H. sapiens*), cow (*B. bovine*), humpback whale (*M. novaeangliae*), and bowhead whale (*B. mysticetus*). The maximum life spans of these organisms range from ∼4 years (mouse, hamster, and rat) to ∼200 years (bowhead whale).

The global analysis of protein turnover rates was conducted using a dynamic (or pulsed) SILAC strategy (4, 9, 17, 18, 29, 51, 52) as illustrated in Figure 1*B* and described in detail in Experimental Procedures. Briefly, cells were cultured at 37 °C and grown to a contact-inhibited quiescent state before isotopic labeling. Typically, in dividing cells, the fractional rate of isotopic labeling is influenced by both degradation rates and cellular proliferation rates (4, 53). By conducting the labeling experiments in a nondividing state, we ensured that variations in cellular proliferation rates of different cultured cells were not influencing our observed labeling kinetics. In addition, conducting the analyses in quiescent cells allowed the measurement of turnover kinetics for stable proteins whose half-lives are significantly longer than the doubling time of the cells (23, 53, 54).

The fractional labeling of tryptic peptides was measured after 2, 4, and 6 days of isotope incorporation with ^13^C-lysine and ^13^C-arginine. We were able to quantify the labeling for more than 6000 peptides mapped to more than 1500 proteins from each species (supplemental Tables S1 and S2). For each protein, data from all corresponding peptides were aggregated and the median fractional labeling at each time point was calculated. In general, the labeling patterns of peptides mapped to the same protein closely mirrored one another (supplemental Fig. S2). For each protein, the median measurements of fractional labeling of mapped peptides at each time point were fit to a single-order exponential equation (assuming first-order kinetics), and degradation rate constants (*k*_*deg*_) were measured (Fig. 1*C*, supplemental Table S3). To assess the precision of the *k*_*deg*_ measurements, we repeated the analysis for fibroblasts isolated from two individual bowhead whales (Fig. 1*D*). The Pearson correlation coefficient for *k*_*deg*_ measurements between these two biological replicates was 0.95. The distribution of *k*_*deg*_ measurements for all identified proteins and for proteins shared between the species are shown in Figure 1*E* and supplemental Fig. S3, respectively.

### Negative Correlation Between Life Span and Protein Degradation Rates

**Fig. 2:**
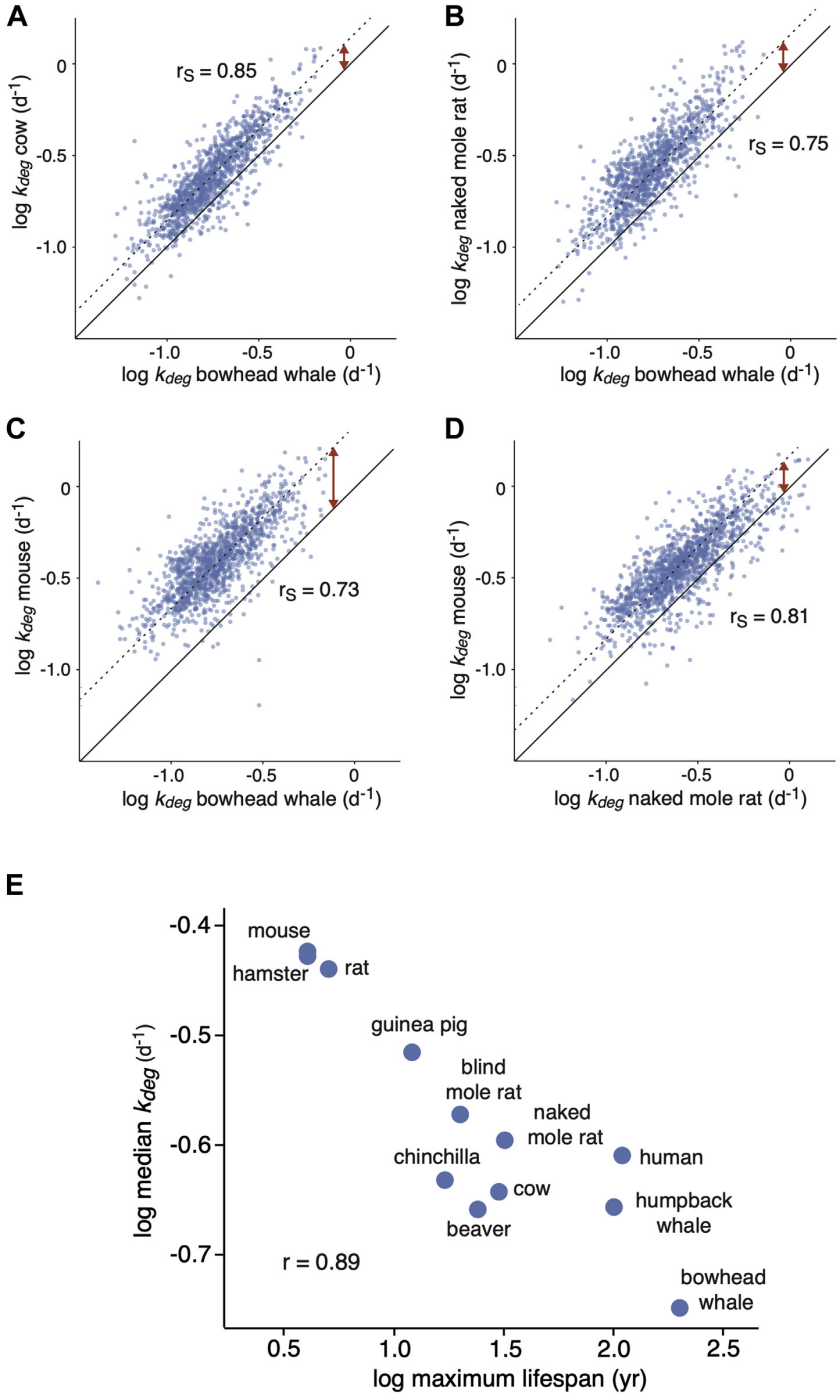
**Cross-species comparison of *k***_***deg***_**measurements.***A*–*D*, pairwise comparisons of *k*_*deg*_ measurements for representative pairs of species with variable life spans and evolutionary divergence times. *Solid lines* indicate lines of identity, and *dotted lines* indicate lines of best fit. *Red arrows* highlight global shifts in distributions of *k*_*deg*_. r_S_ values indicate Spearman rank correlation coefficients. *E*, correlation between species’ median *k*_*deg*_ values and maximal life spans. The r value indicates the Pearson correlation coefficient.

Comparisons of *k*_*deg*_ measurements between different pairs of species indicated relatively strong correlations with Spearman rank correlation coefficient (r_S_) values of all comparisons ranging from 0.63 to 0.87 (supplemental Fig. S4*A*). As expected, *k*_*deg*_ values of closely related species were more correlated than evolutionary distant species (supplemental Fig. S4*B*). For example, the correlation of *k*_*deg*_ measurements between the bowhead whale and cow (55 million years ago divergence, Fig. 2*A*) was greater than that of the bowhead whale and naked mole rat, or the bowhead whale and mouse (87.5 million years ago divergence, Fig. 2, *B* and *C*).

In addition to differences in correlation, pairwise comparisons of species highlighted global shifts in distribution of degradation rates (Fig. 2, *A*–*D*, red arrows). We calculated median degradation rates for proteomes of each species and assessed their correlation to several organismal properties including the maximum life span and body mass. We observed a strong negative correlation between median *k*_*deg*_ values and maximal life span, taking into account all quantified proteins (Fig. 2*E*, r = −0.89) or limiting the analysis to orthologous proteins shared between species (supplemental Fig. S3*B*, r = −0.90). A significant but weaker correlation was observed between *k*_*deg*_ values and body mass (supplemental Fig. S5, r = −0.76 and r = −0.78 for all and shared proteins, respectively). After correcting for the effect of phylogenetic distance (55), the correlation between median degradation rates and the maximal life spans had a two-tailed *p*-value of 0.001, suggesting that these two parameters are independently correlated across a diverse set of mammals. Thus, our data indicate that longer lived organisms generally have slower global protein turnover rates.

### Degradation Rates of Abundant Proteins Are Particularly Diminished in Long-Lived Organisms

**Fig. 3:**
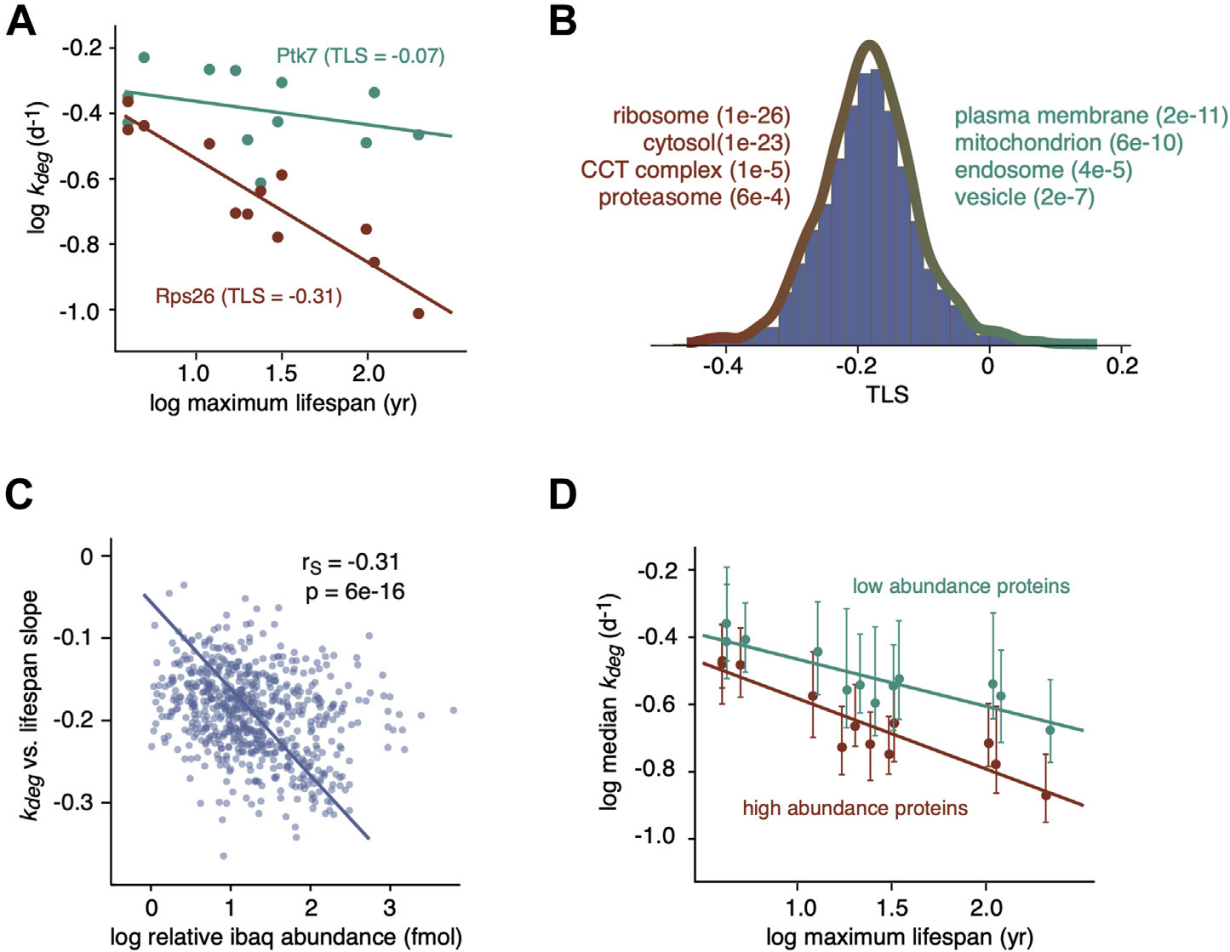
**Differences in protein *k***_***deg***_**values between species are correlated with protein abundance.***A*, correlation between *k*_*deg*_ values of two example proteins (Ptk7 and Rps26) and maximum life spans across species. The turnover–life span slope (TLS) measurements refer to slopes of the log-log plots. *B*, distribution of protein TLS values. Steep and shallow TLS values are most enriched in proteins mapped to *red* and *cyan* GO terms, respectively. *C*, correlation between protein TLS values and abundances (measured in mouse cells). The *line* indicates the line of best fit. The r_S_ and *p*-values indicate the Spearman rank correlation coefficient and significance, respectively. *D*, correlation between median *k*_*deg*_ values and maximum life spans across species for the 500 most (*red*) and least (*cyan*) abundant proteins in the data set. CCT, chaperonin-containing tailless complex; GO, gene ontology.

We next assessed the relationship between organismal life span and degradation rates of individual proteins. We observed that this relationship was variable between proteins. For example, *k*_*deg*_ measurements of orthologues of the kinase Ptk7 were relatively consistent among all analyzed species, whereas *k*_*deg*_ measurements of orthologues of the ribosomal protein Rps26 were significantly reduced in longer lived organisms (Fig. 3*A*). As a quantitative metric of life span dependence, we measured the slope of log *k*_*deg*_ *versus* log life span for individual proteins (Fig. 3*A*, supplemental Table S4). We refer to this parameter as the TLS. The TLS values were calculated for all proteins where *k*_*deg*_ measurements were determined for five or more species. We next determined if proteins with high or low TLS values were enriched in specific GOs (Fig. 3*B*, supplemental Table S5). Among GO terms that were most prevalent within proteins with low TLS values (*i.e.*, had more negative slopes) were cytosolic complexes such as the ribosome, proteasome, and chaperonin-containing tailless complex /T-complex protein ring complex chaperonin, as well as proteins localized to the endoplasmic reticulum (supplemental Fig. S6, *A* and *B*). Conversely, membrane proteins, mitochondrial proteins, and proteins localized to intracellular vesicles such as the endosome had higher TLS values that were closer to zero.

We noted that the protein complexes whose degradation rates were most reduced in long-lived organisms were among the most highly abundant cytosolic proteins in eukaryotic cells (56, 57, 58). To determine if there was a general relationship between protein abundance and TLS, we measured the abundance of each protein within mouse cells using iBAQ (supplemental Table S6) (46). This proteomic methodology provides a proxy for the steady-state level of each protein by measuring the sum of MS peak intensities of all peptides matching the protein normalized by the number of theoretically observable peptides. By spiking in a set of absolute internal standards, we showed that iBAQ measurements were linearly correlated with absolute protein amounts across four orders of magnitude (supplemental Fig. S6*C*). Using this approach, we confirmed that proteins mapped to GO terms with the lowest TLS values were significantly more abundant than proteins mapped to GO terms with higher TLS values (supplemental Fig. S6*D*). Indeed, for individual proteins, there is a significant overall correlation between abundance and TLS (Fig. 3*C*). As a further illustration of this phenomenon, we showed that the TLS values of the 500 most abundant orthologous proteins within our data set were significantly lower than the 500 least abundant proteins (Fig. 3*D*). Together, the data indicate that long-lived organisms may have evolved to specifically reduce turnover rates of highly abundant proteins.

### In Comparison with Mouse Cells, Naked Mole Rat Cells Have Lower Levels of ATP Production

Protein turnover is one of the most energetically expensive processes in eukaryotic cells (59). Thus, the observation that turnover rates of abundant proteins are lower in long-lived organisms suggested that the longevity benefits of slow turnover may be related to reduced energy expenditure. We therefore explored metabolic differences between cells derived from a representative pair of species with divergent life spans and protein turnover rates. We focused these studies on comparisons of mouse and naked mole rat cells as these two rodent species differ widely in life spans and protein turnover rates yet are closely related evolutionarily and have similar body masses. Comparative studies of mice and naked mole rats have been used extensively in the aging field to identify cellular and physiological characteristics associated with longevity (60, 61).

**Fig. 4:**
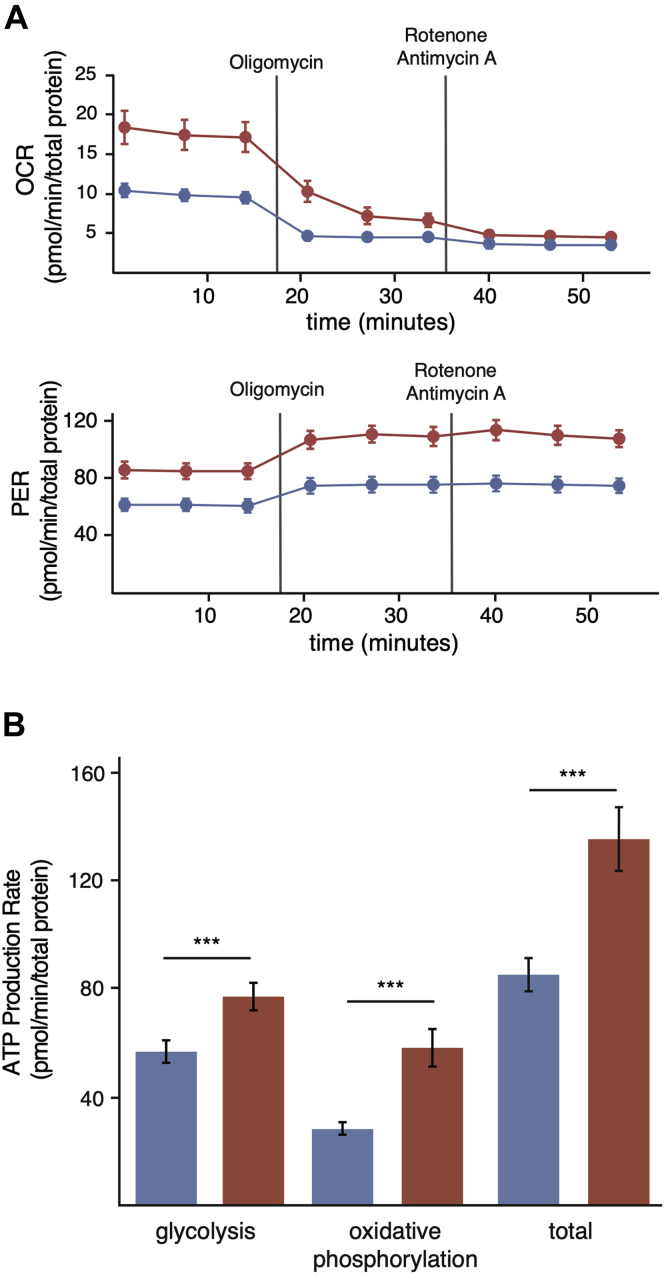
**Differences in ATP production rates between mouse and naked mole rat cells.***A*, rates of oxygen consumption (*top*) and proton efflux (*bottom*) of mouse (*red*) and naked mole rat (*blue*) fibroblasts. *Solid vertical lines* indicate the injection time of the specified inhibitor. *B*, measurements of ATP production from glycolysis and oxidative phosphorylation in mouse (*red*) and naked mole rat (*blue*) cells. Error bars indicate SD. ∗∗∗*p*-value < 0.0005. OCR, oxygen consumption rate; PER, proton efflux rate.

We measured ATP production rates in quiescent mouse and naked mole rat cells using the Seahorse ATP Production Rate assay (62). The experiments provided measurements of OCR and media acidification (PER) in real time. Under quiescent conditions, we observed that mouse cells consume oxygen and acidify the media at a more rapid rate than naked mole rat cells (Fig. 4*A*). The addition of oligomycin, an ATP synthase inhibitor, results in a decrease in the OCR that can be used to calculate the rate of ATP production from oxidative phosphorylation. The addition of a mixture of antimycin A and rotenone, inhibitors of electron transport chain (ETC) complexes I and III, respectively, results in an increase in PER and can be used to calculate the rate of ATP production from glycolysis (62). Using these treatments, we observed that naked mole rat cells produce ATP at a slower rate from both glycolysis and oxidative phosphorylation than mouse cells (Fig. 4*B*).

**Fig. 5:**
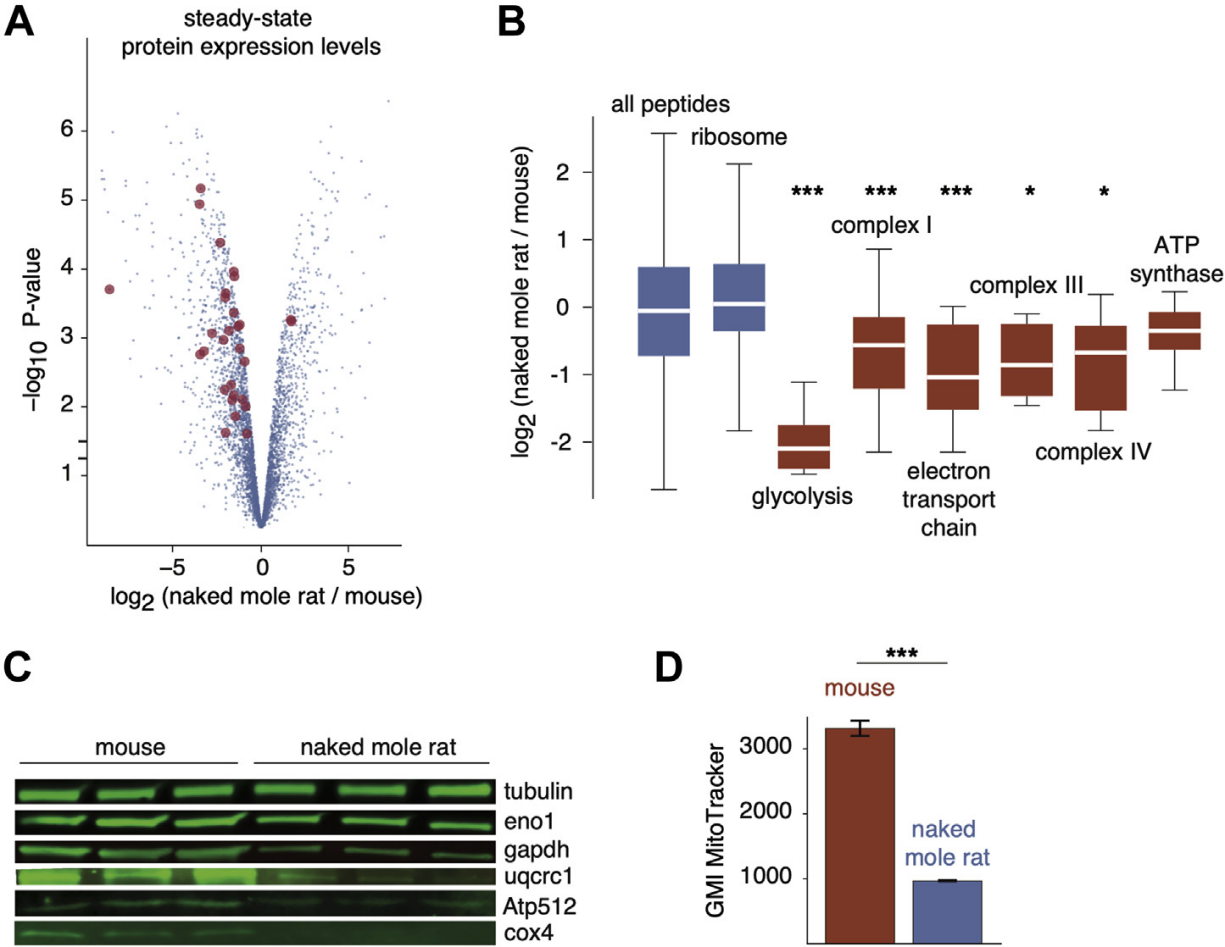
**Proteome-wide differences in steady-state protein levels between mouse and naked mole rat cells.***A*, the volcano plot of the *p*-value *versus* log_2_ ratio of expression levels in naked mole rat and mouse cells. *Blue points* represent all proteins, and *red points* highlight proteins involved in glycolysis and oxidative phosphorylation with significantly altered expression levels. *B*, distribution of log_2_ expression level ratios for specified protein subsets. The box plot representations are as described in Figure 1. *Red boxes* highlight GO terms involved in ATP production. ∗, and ∗∗∗ indicate *p*-values of less than 0.05, 0.005, and 0.0005, respectively, in comparison with the global distribution using the Mann–Whitney *U* test. *C*, western blots of selected proteins from respiratory chain complexes (Uqcrc1, Atp512, Cox4) and glycolysis (Eno1, Gapdh). *D*, measurements of geometric mean intensities of MitoTracker mitochondrial staining of naked mole rat and mouse cells. GO, gene ontology.

To gain insight into potential molecular mechanisms of reduced ATP production rates in naked mole rat cells, we conducted LFQ proteomic experiments to identify proteins with altered steady-state expression levels relative to mouse cells. Quiescent mouse and naked mole rat cells were cultured in three biological replicate experiments, and relative changes in protein levels were analyzed using an LFQ methodology (see Experimental Procedures, Fig. 5, supplemental Table S7). Proteins whose relative expression levels were lower in naked mole rats were most enriched in GO terms related to glycolysis and ETC proteins (Fig. 5*B*, supplemental Fig. S7, supplemental Table S8). Proteins with increased expression levels in naked mole rat cells had comparatively lower levels of enrichment for specific GO terms (supplemental Table S8). The decreased expression levels of glycolytic and ETC proteins were confirmed by Western blots (Fig. 5*C*). These results, in addition to previous results obtained by other groups (63), suggest that lower rates of ATP production in naked mole rat cells may be related to lower expression levels of enzymes that play a role in the two major ATP-producing metabolic pathways in eukaryotic cells (glycolysis and oxidative phosphorylation). These reductions are consistent with reduced total mitochondrial volumes within naked mole rat cells as determined by mitochondrial staining (Fig. 5*D*).

**Fig. 6:**
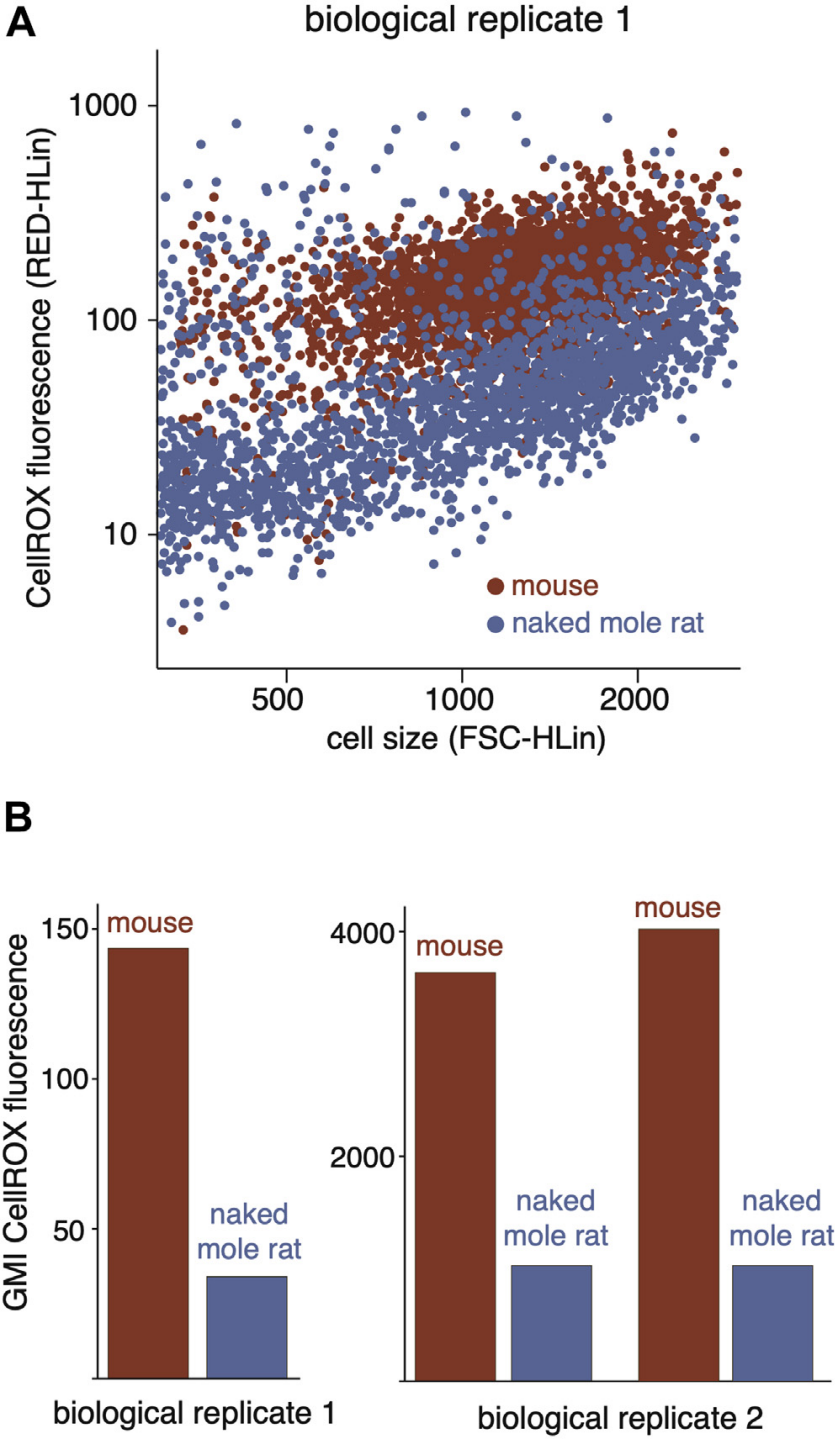
**Differences in cellular ROS levels between mouse and naked mole rat cells.***A*, flow cytometry analysis of CellROX fluorescence (ROS levels) and forward scatter (cell size) for mouse (*red*) and naked mole rat (*blue*) cells. *B*, geometric mean intensities of CellROX fluorescence in mouse (*red*) and naked mole rat (*blue*) cells. Biological replicates represent cell lines cultured from individual organisms. The two pairs of measurements for biological replicates 2 represent measurements of distinct growths of the same cell line. The two biological replicates were measured using different flow cytometers as described in Experimental Procedures. ROS, reactive oxygen species.

### In Comparison With Mouse Cells, Naked Mole Rat Cells Have Lower Levels of ROS Production

Production of ROS is a byproduct of oxidative phosphorylation, and detrimental ROS-induced damage of cellular macromolecules accumulates during the course of aging (64). Given that quiescent naked mole rat and mouse fibroblasts have different rates of protein turnover and ATP production, we assessed whether they also have concordant differences in steady-state ROS levels. We measured cytoplasmic ROS levels using CellROX Orange, a cell-permeable probe whose fluorescence emission is enhanced upon oxidation (65). Quantitation of fluorescence by flow cytometry indicated that under quiescent conditions, mouse cells have significantly higher levels of cytoplasmic ROS than naked mole rat cells, in accordance with previous findings (Fig. 6) (66). The results are consistent with the idea that reduced energy expenditure due to slow protein turnover may be associated with reduced ROS accumulation in long-lived organisms.

### Slow Protein Turnover Does Not Compromise Proteostasis in Naked Mole Rat Cells

**Fig. 7:**
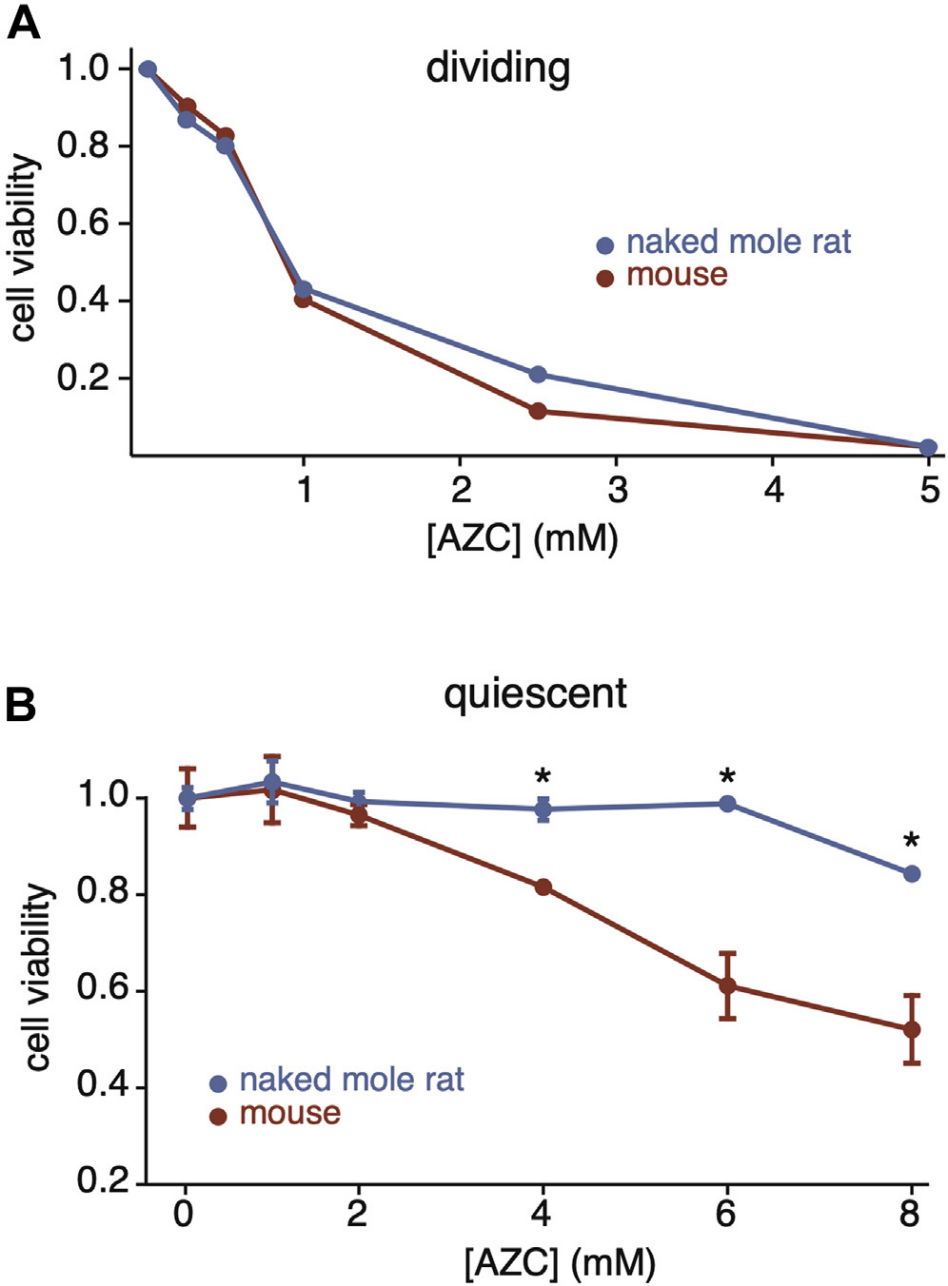
**Cellular survival in the presence of protein misfolding stress.***A*, the response to AZC treatment in dividing mouse (*red*) and naked mole rat (*blue*) cells. Cell viability represents the fraction of live cells in treated cells relative to untreated cells. *B*, the response to AZC treatment in quiescent mouse (*red*) and naked mole rat (*blue*) cells. Cell viability represents the fraction of cells that excluded trypan blue. Error bars indicate SD. ∗*p*-value < 0.05. AZC, azetidine-2-carboxylic acid.

Slow protein turnover may be conducive to long life spans because of concomitant reductions in energy expenditure and ROS accumulation. However, in theory, slow turnover may also have contradictory detrimental effects on proteostasis by reducing the clearance rates of damaged and misfolded proteins. We therefore assessed the ability of naked mole rat and mouse fibroblasts to tolerate proteotoxic stress. To induce proteotoxic stress, we treated cells with L-AZC, a proline analogue that can cause misfolding upon incorporation into proteins and is known to induce a cellular proteotoxic response (67, 68, 69). Mouse and naked mole rat cells were treated with varying concentrations of AZC in proline-containing media for 5 days, and the viability was assessed using trypan blue stain. Under dividing conditions, naked mole rat and mouse cells can tolerate similar doses of AZC before the onset of toxicity (Fig. 7*A*). Interestingly, under quiescent conditions, naked mole rat cells can survive in the presence of higher concentrations of AZC than mouse cells (Fig. 7*B*). Thus, slower protein turnover does not appear to compromise the ability of naked mole rat cells to respond to protein misfolding stress. In fact, under quiescent conditions, naked mole rat cells can tolerate higher concentrations of AZC than mouse cells.

**Fig. 8:**
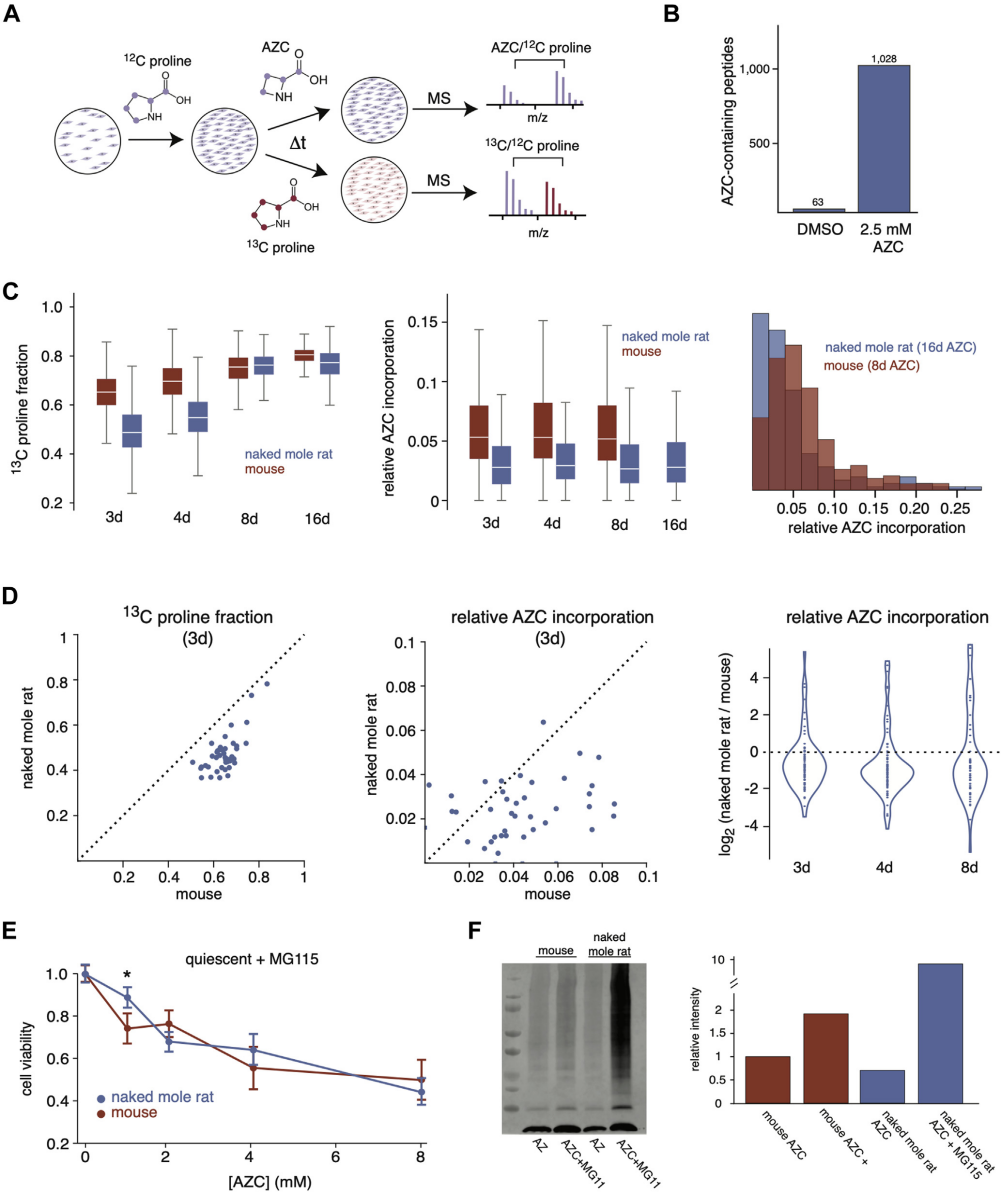
**Accumulation of AZC in the proteomes of mouse and naked mole rat cells.***A*, the experimental design of proteomic experiments for measurement of AZC incorporation. *Blue* and *red* colors indicate ^12^C-proline and either AZC or ^13^C-proline, respectively. *B*, the number of AZC-containing peptides that were quantified in mouse cells treated with either DMSO or 2.5-mM AZC. *C*, incorporation of ^13^C-proline (*left*) and AZC (*center*, *right*) in mouse (*red*) and naked mole rat (*blue*) cells over time. The box plot representations are as described in Figure 1. The histogram to the *right* compares AZC incorporation in naked mole rats after 16 days of treatment with mouse cells after 8 days of treatment. *D*, comparisons of ^13^C-proline (*left*) and AZC (*center*, *right*) incorporation for individual peptides in mouse and naked mole rat cells. For pairwise comparisons (*left, middle*), peptides that could be quantified in both mouse and naked mole rat cells at 3 d were compared. Similar plots for other time points are shown in supplemental Fig. S8. For the violin plot (*right*), log_2_ ratios of incorporation in mouse and naked mole rat cells are plotted for all peptides for which AZC incorporation could be measured in both species. *E*, the response to AZC and MG115 treatment in quiescent mouse (*red*) and naked mole rat (*blue*) cells. Cells were treated with 5-μM MG115 proteasome inhibitor and varying concentrations of AZC for 4 days. Cell viability represents the fraction of cells that excluded trypan blue, normalized to viability when treated solely with MG115 within each species. Error bars represent SD. ∗*p*-value < 0.05. *F*, accumulation of ubiquitinated proteins upon proteasomal inhibition and AZC treatment in mouse and naked mole rat cells. Cells were treated with 5-μM MG115 proteasome inhibitor and 2.5-mM AZC for 4 days, and the extracts were analyzed by Western blots using an anti-ubiquitin antibody. The right plot represents the quantification of the Western blot. The level of ubiquitin in MG115-treated samples for each species was normalized to the level of ubiquitin in the just AZC-treated mouse cells. AZC, azetidine-2-carboxylic acid.

The enhanced ability of naked mole rat cells to tolerate AZC may be due to either lower levels of AZC incorporation into their proteomes or their ability to survive in the presence of higher levels of intracellular AZC-containing proteins. To distinguish between these two possibilities, we used a proteomic approach to quantify the levels of AZC-containing proteins in mouse and naked mole rat cells after exposure to AZC in the growth media (Fig. 8*A*). Quiescent cells were treated with a nonlethal dose of AZC in proline-containing media, and extracts were collected for LC-MS/MS analysis at varying exposure times. An initial pilot experiment indicated that AZC-containing peptides can be identified and quantified in AZC-treated mouse cells with a low false-positive identification rate (Fig. 8*B*). The kinetic MS analyses quantified the peak intensities of AZC-containing peptides normalized with respect to their proline-containing counterparts. As a control, we also dosed cells with a benign isotopically labeled variant of proline (^13^C-proline) and quantified its fractional incorporation as a function of time. Together, the experiments allowed us to compare the accumulation of a benign and a proteotoxic analogue of proline in the proteomes of these two species.

The results indicated that mouse cells accumulated benign ^13^C-prolines in their proteomes faster than naked mole rat cells (Fig. 8*C*, supplemental Table S9). This observation was consistent with the faster rate of turnover observed in mouse cells using isotopically labeled lysines and arginines (Fig. 2*D*). After 8 days of labeling with ^13^C-proline, the levels of incorporation were near 100% for both the mouse and naked mole rat as their proteomes had almost fully turned over during this time span. In contrast, levels of AZC incorporation remained significantly lower in naked mole rat cells even after 8 days of labeling. Indeed, naked mole rat cells exposed to AZC for 16 days contained less AZC in their proteomes than mouse cells exposed to AZC for 8 days (Fig. 8*C*). Unlike naked mole rat cells, AZC incorporation could not be measured for mouse cells at 16 days because of the onset of toxicity and cell death at this time. The results suggest that the ability of naked mole rat cells to tolerate higher levels of AZC in the media is related to reduced accumulation of AZC-containing proteins in their proteomes.

We reasoned that there are two possible explanations for why AZC-exposed naked mole rat cells accumulate less AZC in their proteomes in comparison with mouse cells. First, the ability of AZC to compete with proline during the process of protein synthesis (*e.g.*, aminoacylation of tRNAs or incorporation into nascent polypeptides during translation) may be less efficient in naked mole rat cells. Second, naked mole rat cells may have an enhanced ability to specifically detect and clear AZC-containing and potentially damaged proteins once they have been synthesized. Our data appear more compatible with the second explanation as differences in levels of AZC incorporation between mouse and naked mole rat cells are highly variable between orthologous proteins (Fig. 8*D*). If naked mole rat cells had a reduced ability to aminoacylate tRNAs with AZC, or to use tRNAs charged with AZC during translation, we would expect a generally uniform reduction in AZC incorporation for all observed proteins. Conversely, if misfolded AZC-containing proteins are cleared more efficiently in naked mole rat cells, we would expect variable levels of AZC incorporation for different proteins (in comparison with mouse cells) as the effect of AZC incorporation on misfolding would be dependent on its sequence context. In other words, AZCs incorporated in different proteins, or different parts of the same protein, are expected to have variable repercussions on folding, and hence different effects on clearance of the protein. When comparing AZC incorporation for identical peptides between mouse and naked mole rat cells at different time points, we observe that the decrease in AZC incorporation in naked mole rat cells is highly variable between peptides (Fig. 8*D*, supplemental Fig. S8). These data suggest that the reduced accumulation of AZC-containing proteins in naked mole rat cells may be related to an enhanced ability to specifically detect and clear damaged proteins.

### In Comparison With Mouse Cells, Naked Mole Rat Cells Have Higher Rates of Proteasomal Clearance of Ubiquitinated Proteins

The best characterized mechanism for selective degradation of misfolded proteins in eukaryotic cells is the UPS (70). To determine if the enhanced ability of naked mole rat cells to tolerate AZC is dependent on proteasomal degradation, we monitored the viability of these cells after AZC treatment and partial proteasomal inhibition by the inhibitor MG115 (Fig. 8*E*). The results indicate that the enhanced ability of naked mole rat cells to tolerate AZC treatment is nullified upon proteasomal inhibition. We further showed that in comparison with mouse cells, AZC-treated naked mole rat cells have lower steady-state levels of ubiquitinated proteins but accumulate higher levels of ubiquitinated proteins upon proteasomal inhibition (Fig. 8*F*, supplemental Fig. S9). The results suggest that after AZC treatment, the proteolytic flux of ubiquitinated proteins through the proteasome is faster in naked mole rat cells than mouse cells. These results are consistent with previous studies showing that naked mole rat cells possess relatively high proteasome activity (71). Robust degradation of damaged ubiquitinated proteins through the UPS provides a mechanistic explanation for the enhanced ability of naked mole rat cells to selectively clear AZC-containing proteins and survive proteotoxic stress despite having slow rates of basal protein turnover.

## Discussion

To the best of our knowledge, the data presented in this article represent the largest cross-species comparison of proteome turnover rates carried out to date. The *in vitro* analyses were conducted in quiescent dermal fibroblasts cultured from 12 mammalian species with widely divergent life spans. The results highlighted a negative correlation between global protein turnover rates and organismal longevity. The generality of this correlation in other organisms, tissues, cell types, and environmental conditions remains to be determined. Nonetheless, the data provide a metabolic rationale for why it may be advantageous for longer lived organisms to maintain relatively slow rates of protein turnover.

Protein turnover is one of the most energetically demanding cellular processes in living organisms, accounting for as much as 25% of the total energy expenditure in some species (59). Thus, slower protein turnover rates can translate into diminished ATP demand and a concomitant reduction in the production of damaging ROS over the course of a long life span. A number of observations in this study are consistent with this idea. First, reductions in protein degradation rates in long-lived organisms are most pronounced for highly abundant proteins whose fractional turnover demands relatively more energy. Second, comparative analyses of mouse and naked mole rat cells (representatives of short-lived and long-lived species, respectively) demonstrate that the latter have slower rates of ATP production and lower expression levels of metabolic enzymes involved in glycolysis and oxidative phosphorylation. Third, naked mole rat cells have lower steady-state ROS levels than mouse cells.

Although our analyses are necessarily correlative and do not directly demonstrate causation, the data are consistent with the idea that slower rates of protein turnover in longer lived organisms contribute to longevity by reducing energy demand and ROS production. However, we cannot rule out the possibility that diminished protein turnover is a tangential consequence of reduced energy expenditure and ROS production in the long-lived organisms analyzed in this study. For example, it has been argued that selection pressures imposed by the high-arctic habitat have favored animals such as the bowhead whale with long life spans (72). To sustain low metabolic rates consistent with limitations of their habitats, these animals may have evolved mechanisms to reduce turnover rates of abundant proteins.

Our data also indicate that slow protein turnover does not necessarily diminish tolerance for protein misfolding stress in long-lived organisms. Indeed, under quiescent conditions, we show that naked mole rat cells have a higher tolerance for AZC, an amino acid analogue that results in misfolding upon incorporation into proteins. The data suggest that this tolerance may be related to the enhanced ability of naked mole rat cells to selectively degrade misfolded AZC-containing proteins. These observations are consistent with enhanced proteostasis in naked mole rat cells that have been reported in previous studies (71, 73).

How is it possible that naked mole rat cells have slow rates of basal protein turnover, yet have an enhanced ability to clear damaged proteins? It has long been appreciated that the turnover of the steady-state protein pool in a cell is largely stochastic and follows single-exponential first-order kinetics (22, 23). This trend has been largely confirmed by more recent proteome-wide analyses of turnover (4, 9, 17, 18, 29, 46, 52). From the perspective of total protein flux, it appears that for a given protein, any one molecule is as likely to be degraded than any other regardless of its age (elapsed time since its synthesis). The exception to this rule appears to be a fraction of newly synthesized proteins that are rapidly degraded immediately after translation and do not contribute to the steady-state protein pool in a cell (74). Thus, it appears that the vast majority of proteins that are present in a cell are not degraded because they have become damaged over time but rather because their intrinsic properties have predetermined their basal half-lives. Nonetheless, protein damage is a common and dangerous occurrence in cells, and a number of sophisticated mechanisms have evolved for specific detection and removal of modified proteins (3, 24). In eukaryotic cells, these mechanisms include the UPS and selective autophagy (28, 37, 70, 75). Thus, the total flux of proteins in a cell can be considered as having two components: nonselective basal turnover and selective clearance of damaged proteins. The former constitutes the larger component of total protein flux and is the rate that is typically measured by isotopic labeling experiments that quantify the overall fractional labeling of proteins over time.

Considering these two modes of protein flux, enhanced removal of damaged proteins can be achieved by increasing the rate of nonselective basal turnover or deployment of more sophisticated surveillance systems for selective detection and clearance of aberrantly modified proteins. The former strategy is significantly more energy intensive and is likely to be associated with higher rates of ROS generation and associated damage over an extended life span. Whereas rapid nonselective basal turnover may represent a viable proteostatic strategy for short-lived organisms, their associated costs in terms of ROS generation may not be conducive to longer life spans. Thus, there may be selective evolutionary pressure for longer lived organisms to lower rates of basal turnover and, in lieu of reduced total protein flux, evolve more efficient mechanisms for selective clearance of damaged proteins. Consistent with this idea, our data indicate that long-lived naked mole rats have the ability to rapidly degrade misfolded proteins through the UPS while maintaining a relatively slow basal rate of constitutive protein turnover.

The distinction between selective and nonselective protein clearance may also provide an explanation for the apparent contradiction in the relationship between turnover rates and longevity that has been observed in different model systems (5). In general, it appears that treatments and genetic modifications that extend the lives of invertebrates such as *C. elegans* and *D. melanogaster* model systems result in faster rates of total protein turnover (31, 39, 40, 41). Conversely, in mammalian models, life-extending treatments and genetic modifications are associated with a slower protein turnover (42, 43, 44, 45). If nonselective protein turnover plays a more prominent role in maintaining proteostasis in shorter-lived organisms, it is understandable why extending life span may require faster rates of total protein flux. Conversely, in longer-lived mammalian systems, increased ROS generation over time represents a greater impediment to longevity, and life span extension is associated with the lower energetic demands of a slow protein turnover. In summary, our results provide support for the idea that the beneficial effects of protein turnover on proteostasis and longevity are mitigated by associated metabolic costs and liabilities.

## Data Availability

All raw and processed data are available in included Supplemental Tables (see below) and at ProteomeXchange Consortium *via* the PRIDE database (accession number PXD018325).

## Conflict of interest

The authors declare no competing interests.
