# Supplementary material for: Interspecies Differences in Proteome Turnover Kinetics Are Correlated With Life Spans and Energetic Demands
Source: Mol Cell Proteomics. 2021 Jan 7;20:100041. doi: 10.1074/mcp.RA120.002301 (PMC7950207; doi:10.1074/mcp.RA120.002301)
Supplement: Supplemental Data 2 [file mmc3.pdf]

ASSOCIATED CONTENT

Spectral search results and quantitations, dynamic SILAC rate measurements, TLS measurements and gene ontology analyses are provided in tabular format in the supplementary tables.

Supplemental Figures

Figure S1. Summary of experiments

Figure S2. Peptide-level comparisons of fractional labeling in dynamic SILAC experiments

Figure S3. Cross-species correlations of protein k_{deg} measurements decrease with increasing evolutionary divergence time.

Figure S4. Distribution of k_{deg} measurements and their correlations with lifespan for the subset of peptides shared among all species

Figure S5. Correlation of k_{deg} measurements with body mass

Figure S6. Cross-species differences in k_{deg} are correlated with protein abundance

Figure S7. GO term enrichment analysis or protein level differences in mouse and naked mole rat cells

Figure S8. 13C proline and AZC incorporation in mouse and naked mole rat cels

Figure S9. Ubiquitination in response to AZC in mouse and naked mole rat cells

Supplemental Tables

Table S1. Coverage of dynamic SILAC experiments (this document)

Table S2. Dynamic SILAC LC/MS/MS data (TableS2.xlsx)

Table S3. $k_{degradation}$ measurements (TableS3.xlsx)

Table S4. Turnover-lifespan slope (TLS) measurements (TableS4.xlsx)

Table S5. Turnover-lifespan slope (TLS) GO enrichment (TableS5.xlsx)

Table S6. iBAQ abundance data (TableS6.xlsx)

Table S7. Mouse vs. naked mole rat LC-MS/MS label free quantitation (LFQ) data (TableS7.xlsx)

Table S8. Mouse vs. naked mole rat LC-MS/MS label free quantitation (LFQ) GO enrichment (TableS8.xlsx)

Table S9. AZC incorporation LC-MS/MS data (TableS9.xlsx)

(Note: Tables S2-S9 are available as individual Excel files with additional descriptions contained within each file)

Accession Numbers

All raw and processed data are available at ProteomeXchange Consortium via the PRIDE database (accession number: PXD018325)

Supplementary Figures

Analyzed Species	Method	Measured parameter
Mouse, Rat, Hamster, Bline Mole Rat, Beaver, Chinchila, Guinea Pig, Naked Mole Rat, Human, Bowhead Whake, Humpback Whale, Cow	Dynamic SILAC	Protein turnover rates
Mouse vs. Naked Mole Rat	Label-free quantitation and iBAQ	Protein levels
	Seahorse assay	ATP production rates
	ROS-specific dyes & flow cytometry	Intracellular ROS levels
	Viability in presence of AZC	Tolerance of proteotoxic stress
	Accumulation of AZC-containing proteins	Accumulation of damaged proteins

Figure S1

Figure S1. Summary of experiments. The table lists the methods and corresponding measured parameters in cross-species experiments reported in the manuscript. Blue listings correspond to proteome-wide analyses and black listings correspond to low throughput biochemical and cell-based studies.

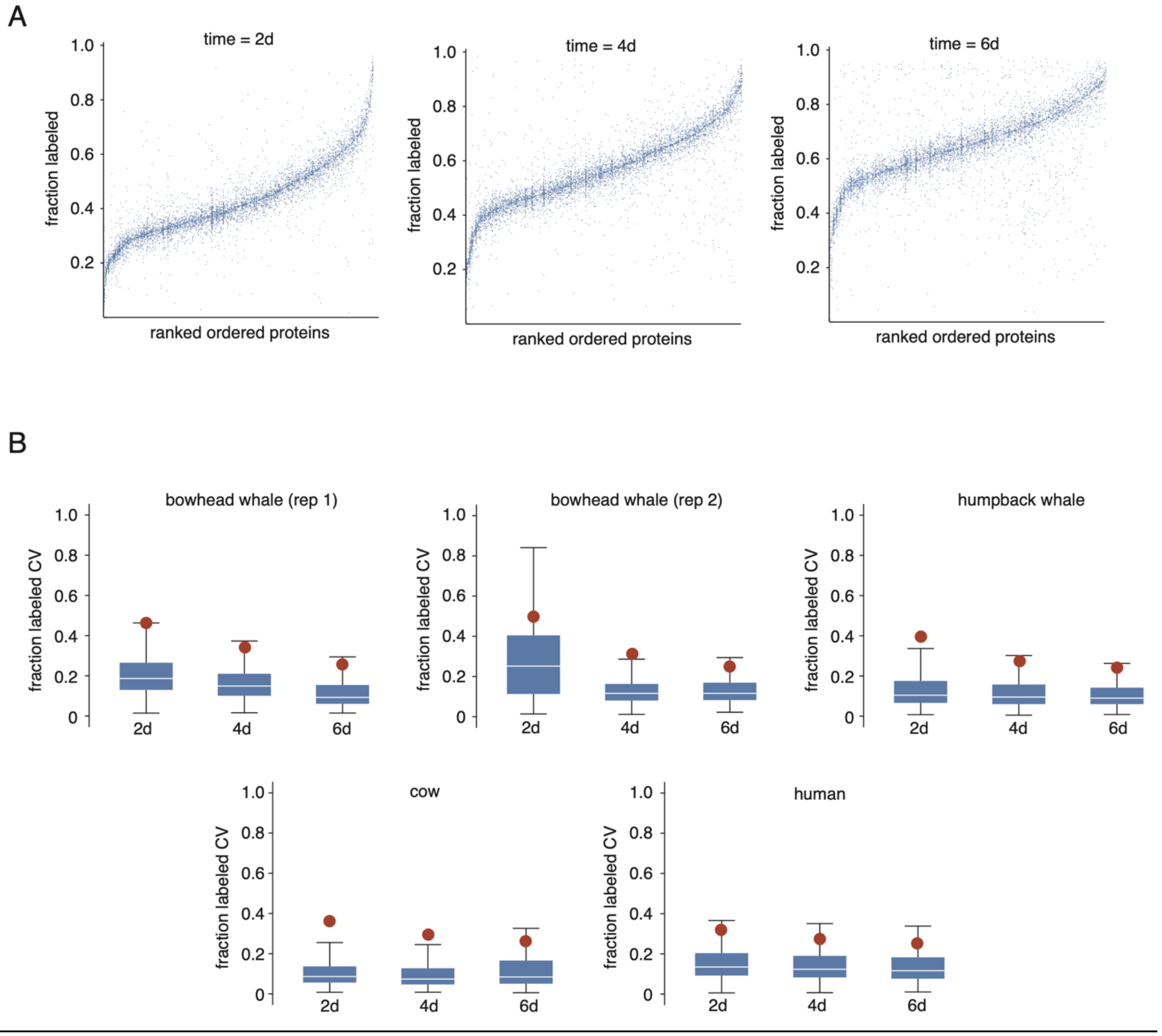
A
time = 2d
time = 4d
time = 6d
fraction labeled
ranked ordered proteins
B
bowhead whale (rep 1)
bowhead whale (rep 2)
humpback whale
cow
human
fraction labeled CV
2d
4d
6d

Figure S2

Figure S2. Peptide-level comparisons of fractional labeling in dynamic SILAC experiments. A. Rank-distribution plots showing the fractional labeling of peptides matched to each protein within the proteome at different time-points. Vertical columns of blue points on the plot represent data for peptides matched to specific proteins. The plots generated for human data are shown as an example. Note that the range of measured fractional labeling for peptides within each protein is significantly narrower than the entire range of all measured peptides within the proteome. Plots from other species show similar trends. B. Box plots indicate the range of coefficient of variations (CVs) for measured fractional labeling of peptides mapped to the same protein. The dots indicate the CV for all peptides at a given time-point. Note that the intra-protein CV of peptide fractional labeling measurements are generally lower than the fractional labeling CV of all peptides in a dataset. Similar analyses for rodent species are presented in Swovick et al (1).

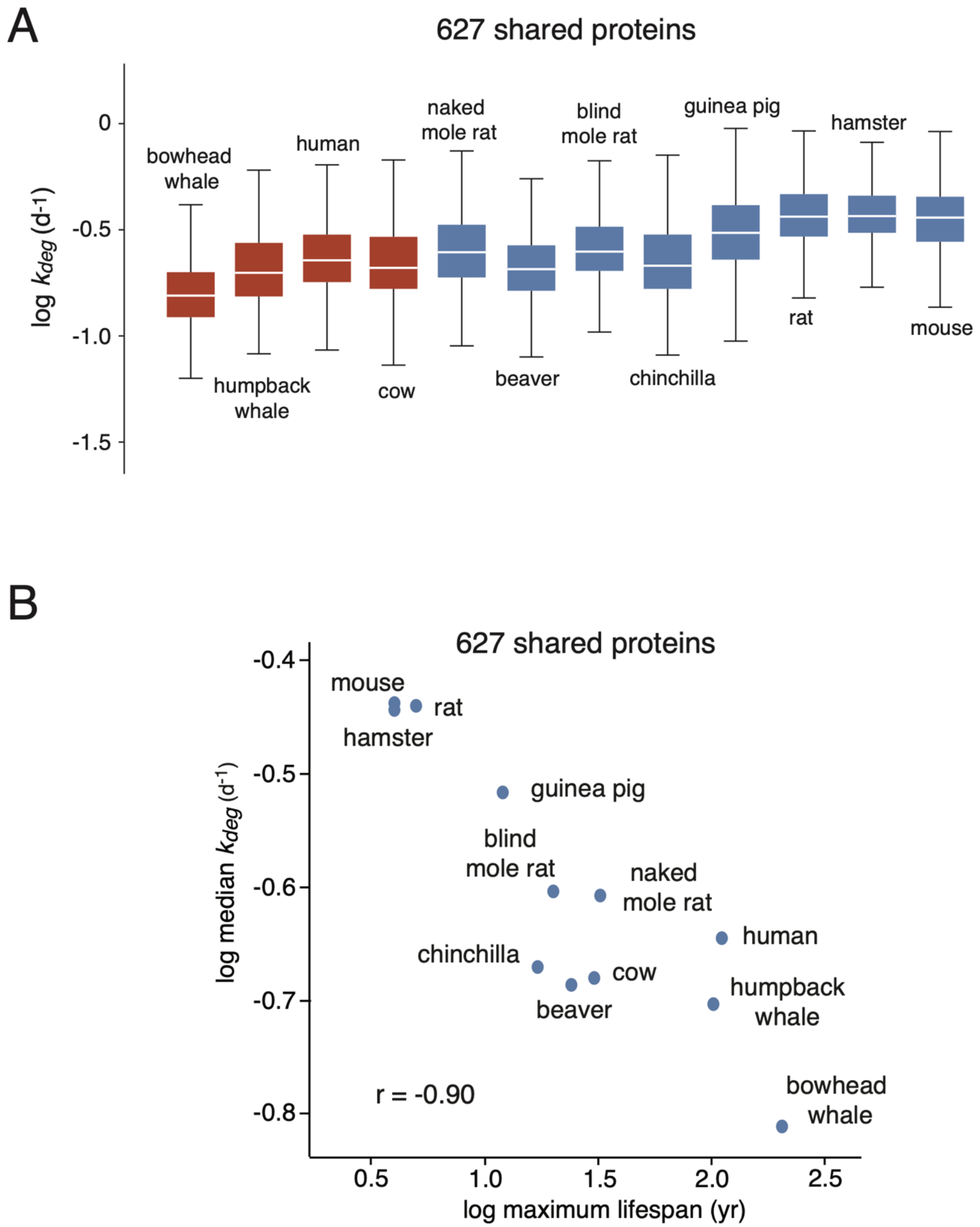
A
627 shared proteins
log k_{deg} (d-1)
0
-0.5
-1.0
-1.5
bowhead whale
humpback whale
human
cow
naked mole rat
beaver
blind mole rat
chinchilla
guinea pig
rat
hamster
mouse
B
627 shared proteins
log median k_{deg} (d-1)
-0.4
-0.5
-0.6
-0.7
-0.8
mouse
rat
hamster
guinea pig
blind mole rat
naked mole rat
human
chinchilla
cow
beaver
humpback whale
bowhead whale
r = -0.90
0.5
1.0
1.5
2.0
2.5
log maximum lifespan (yr)

Figure S3

Figure S3. Distribution of k_{deg} measurements and their correlations with lifespan for the subset of peptides shared among all species. A. Distribution of k_{deg} values for shared 627 orthologous proteins quantified in all species. Red boxes indicate newly generated data while blue boxes indicate data generated by Swovick et al (1). B. Correlation of median k_{deg} values with maximal lifespans for shared 627 orthologous proteins quantified in all species. C. Correlation of median k_{deg} values with adult body mass for all measured proteins (left) and 627 orthologous proteins quantified in all species (right).

A

	bowhead whale	cow	humpback whale	blind mole rat	naked mole rat	beaver	hamster	chinchilla	guinea pig	mouse	rat
**cow**	55 (0.85)										
**humpback whale**	27 (0.79)	55 (0.71)									
**blind mole rat**	87.5 (0.81)	87.5 (0.79)	87.5 (0.74)								
**naked mole rat**	87.5 (0.79)	87.5 (0.79)	87.5 (0.69)	80 (0.79)							
**beaver**	87.5 (0.73)	87.5 (0.73)	87.5 (0.71)	66 (0.73)	70 (0.8)						
**hamster**	87.5 (0.8)	87.5 (0.83)	87.5 (0.67)	45 (0.84)	70 (0.82)	66 (0.77)					
**chinchilla**	87.5 (0.79)	87.5 (0.8)	87.5 (0.72)	70 (0.78)	48 (0.81)	70 (0.76)	70 (0.78)				
**guinea pig**	87.5 (0.78)	87.5 (0.79)	87.5 (0.62)	70 (0.76)	48 (0.85)	70 (0.74)	70 (0.82)	43 (0.81)			
**mouse**	87.5 (0.75)	87.5 (0.76)	87.5 (0.65)	45 (0.81)	70 (0.8)	66 (0.74)	26 (0.85)	70 (0.73)	70 (0.8)		
**rat**	87.5 (0.8)	87.5 (0.79)	87.5 (0.64)	45 (0.85)	70 (0.81)	66 (0.71)	26 (0.87)	70 (0.75)	70 (0.81)	18 (0.85)	
**human**	87.5 (0.82)	87.5 (0.79)	87.5 (0.83)	80 (0.85)	80 (0.76)	80 (0.73)	80 (0.78)	80 (0.75)	80 (0.72)	80 (0.77)	80 (0.78)

B

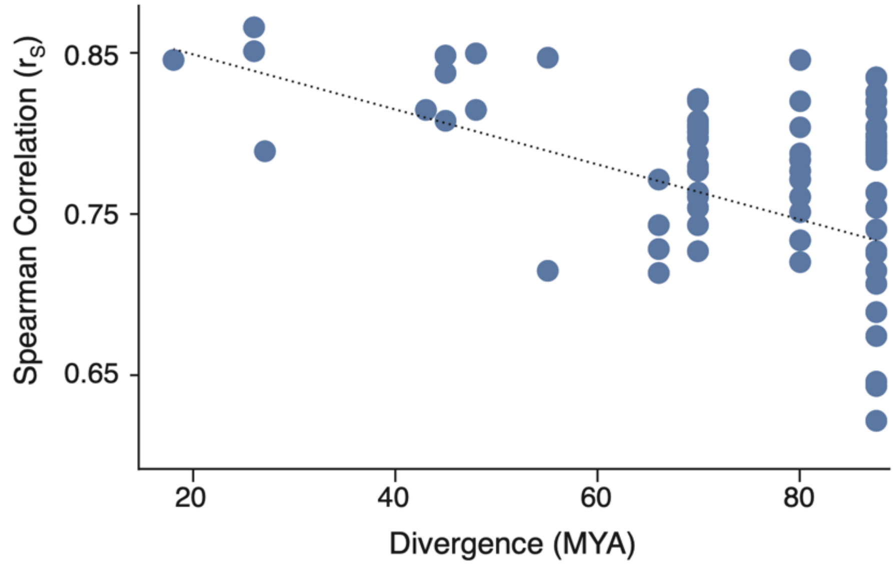

Figure S4

Figure S4. Cross-species correlations of protein k_{deg} measurements decrease with increasing evolutionary divergence times. A. Divergence times in millions of years (top number) and spearman rank correlation coefficients of k_{deg} values (bottom number) for pairs of species. B. Comparison of spearman rank correlation coefficients and divergence times for pairs of species. Dotted line represents the line of best fit.

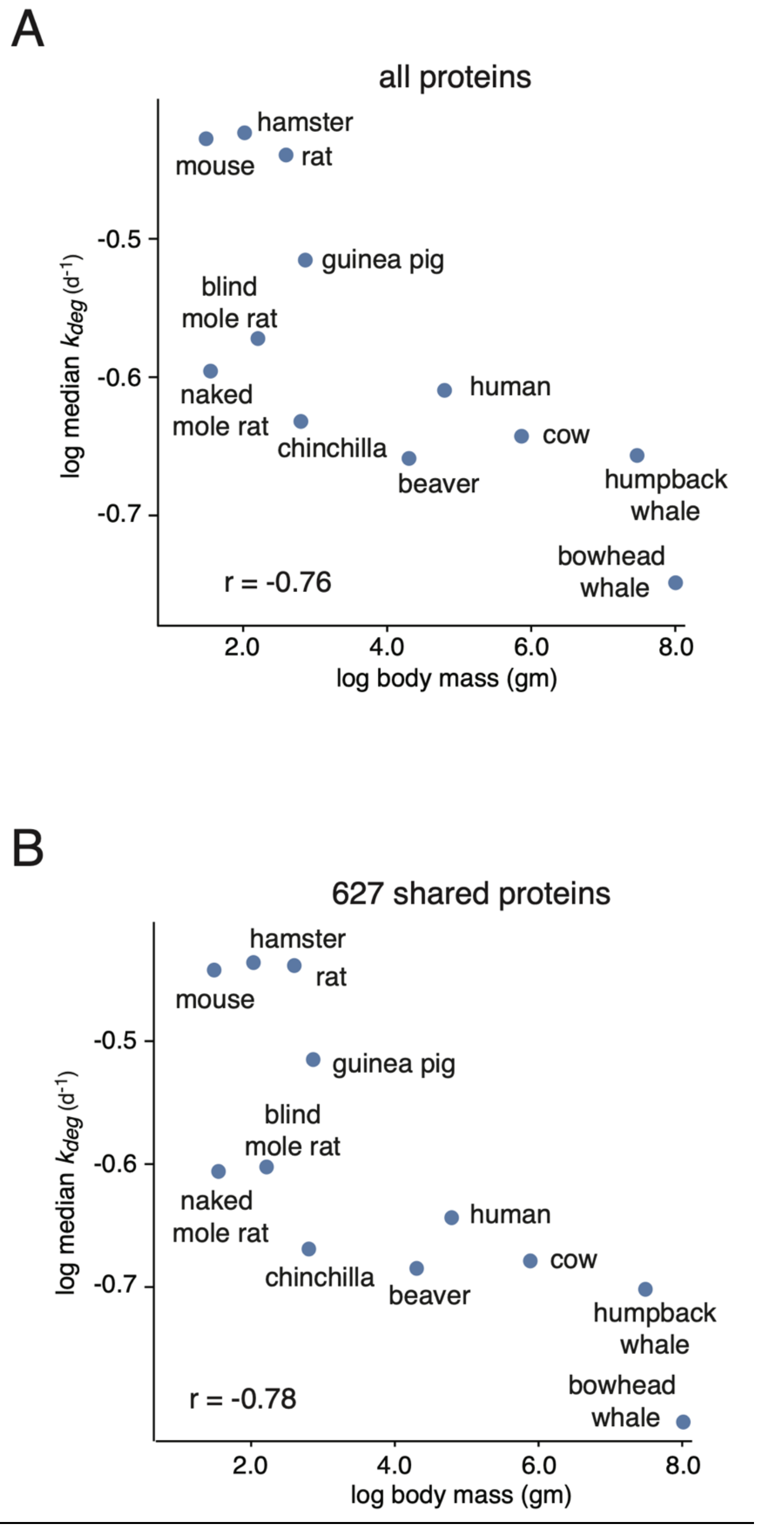
A
all proteins
hamster
mouse
rat
guinea pig
blind
mole rat
naked
mole rat
human
chinchilla
cow
beaver
humpback
whale
bowhead
whale
r = -0.76
-0.5
-0.6
-0.7
log median k_{deg} (d^{-1})
2.0
4.0
6.0
8.0
log body mass (gm)
B
627 shared proteins
hamster
rat
mouse
guinea pig
blind
mole rat
naked
mole rat
human
chinchilla
cow
beaver
humpback
whale
bowhead
whale
r = -0.78
-0.5
-0.6
-0.7
log median k_{deg} (d^{-1})
2.0
4.0
6.0
8.0
log body mass (gm)

Figure S5

Figure S5. Correlation of *k_{deg}* measurements with body mass. Correlation of median kdeg values with adult body mass for all measured proteins (A) and 627 orthologous proteins quantified in all species (B).

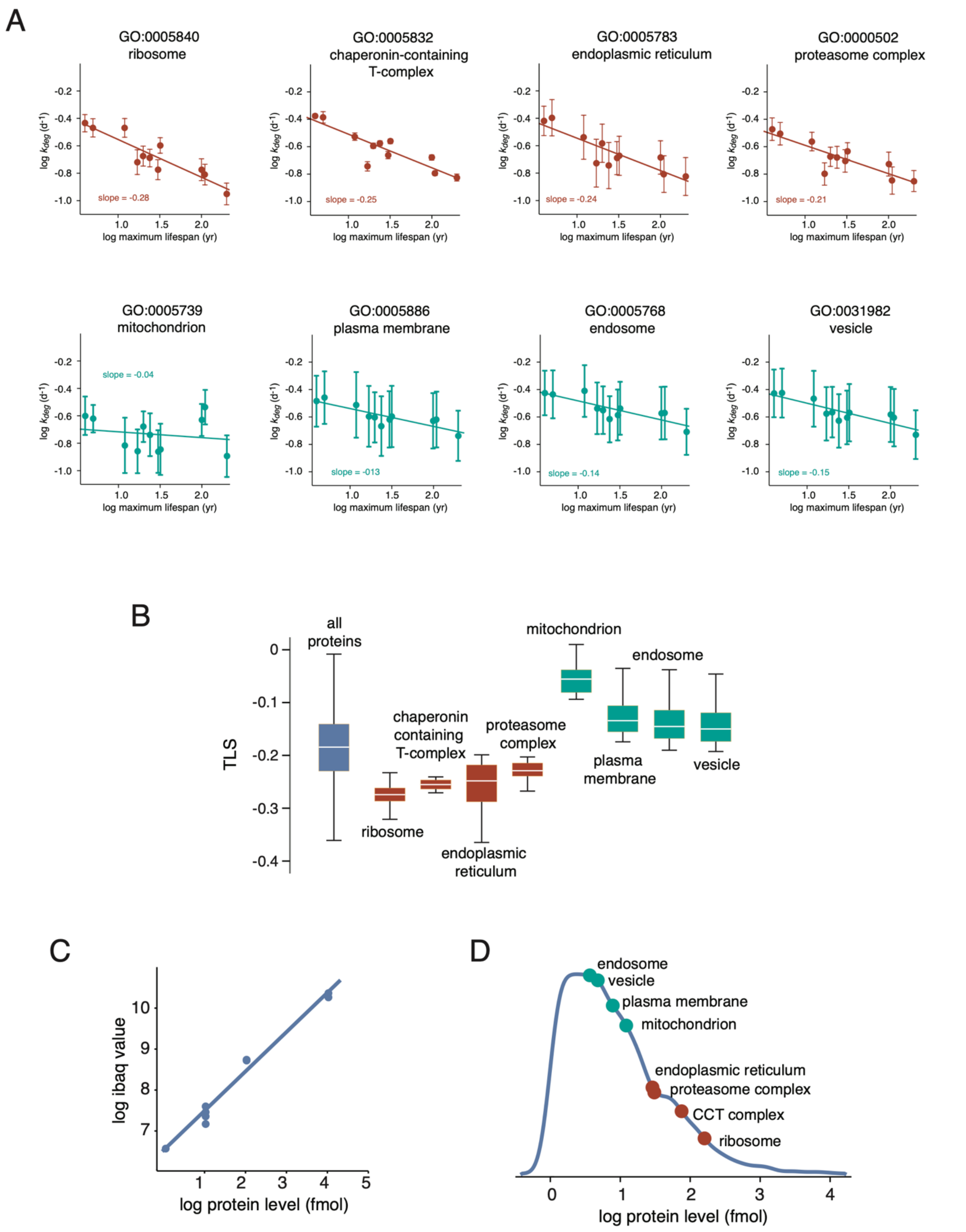
A
GO:0005840
ribosome
slope = -0.28
GO:0005832
chaperonin-containing
T-complex
slope = -0.25
GO:0005783
endoplasmic reticulum
slope = -0.24
GO:0000502
proteasome complex
slope = -0.21
GO:0005739
mitochondrion
slope = -0.04
GO:0005886
plasma membrane
slope = -013
GO:0005768
endosome
slope = -0.14
GO:0031982
vesicle
slope = -0.15
log kdeg (d-1)
log maximum lifespan (yr)
B
TLS
all
proteins
ribosome
chaperonin
containing
T-complex
endoplasmic
reticulum
proteasome
complex
mitochondrion
plasma
membrane
endosome
vesicle
C
log ibaq value
log protein level (fmol)
D
endosome
vesicle
plasma membrane
mitochondrion
endoplasmic reticulum
proteasome complex
CCT complex
ribosome
log protein level (fmol)

Figure S6

Figure S6. Cross-species differences in k_{deg} are correlated with protein abundance

A. k_{deg} of proteins mapped to specific GO terms in different species as a function of maximum lifespan. Red and cyan plots highlight GO terms that have steep and shallow slopes (TLS values), respectively. Error bars represent stand deviation of k_{deg} measurements and lines represent lines of best fit. B. Distribution of TLS values for GO terms shown in A. Box plot representations are as described in Figure 1. C. iBAQ protein abundance calculations in mouse cells. (Left) Standard curve from spiked-in UPS2 standards in iBAQ experiment. (Right) Distribution of absolute protein levels in mouse cells. Indicated are the median abundances for GO terms listed in A and B.

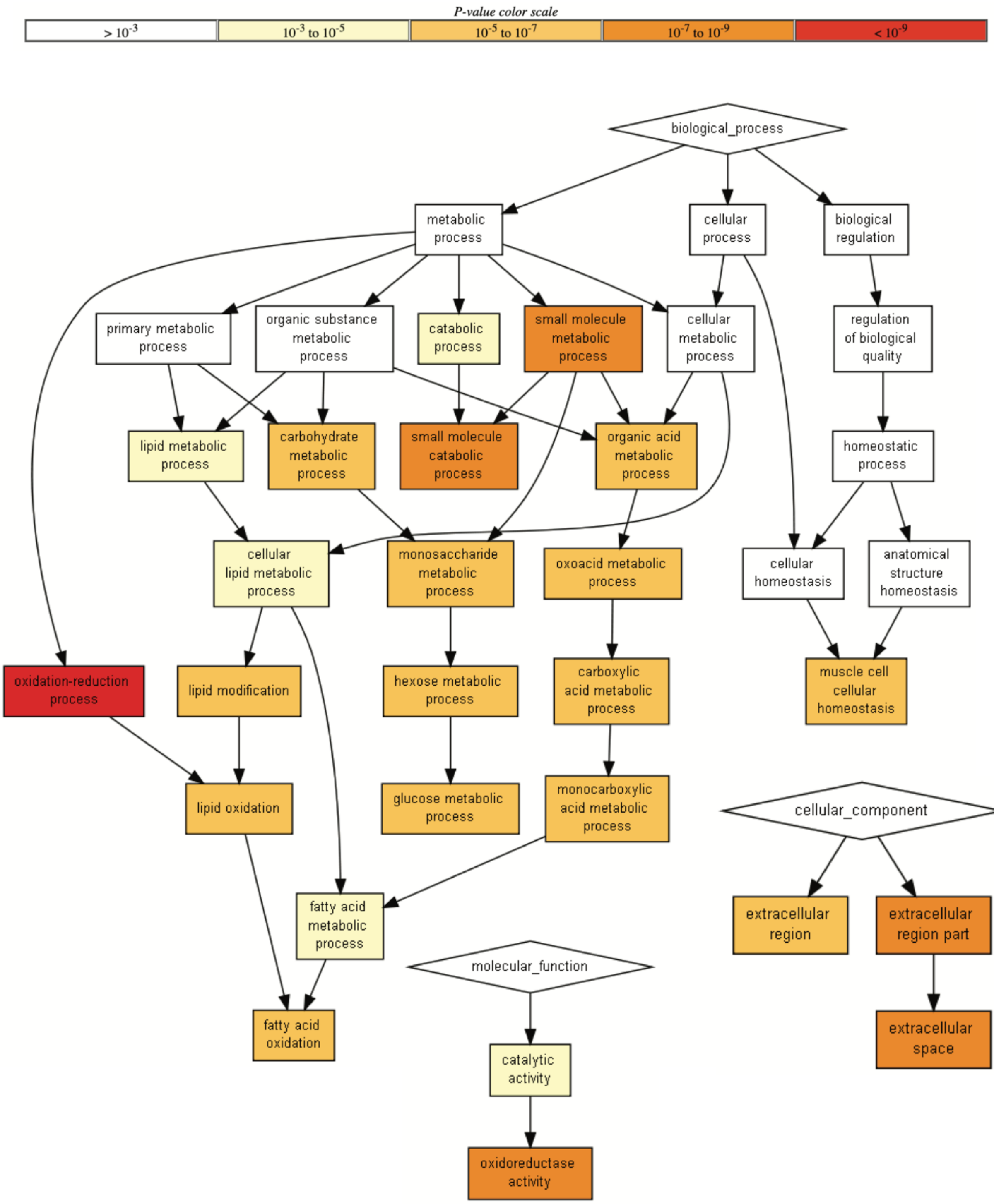
P-value color scale
> 10^{-3}
10^{-3} to 10^{-5}
10^{-5} to 10^{-7}
10^{-7} to 10^{-9}
< 10^{-9}
biological_process
metabolic process
cellular process
biological regulation
primary metabolic process
organic substance metabolic process
catabolic process
small molecule metabolic process
cellular metabolic process
regulation of biological quality
lipid metabolic process
carbohydrate metabolic process
small molecule catabolic process
organic acid metabolic process
homeostatic process
cellular lipid metabolic process
monosaccharide metabolic process
oxoacid metabolic process
cellular homeostasis
anatomical structure homeostasis
oxidation-reduction process
lipid modification
hexose metabolic process
carboxylic acid metabolic process
muscle cell cellular homeostasis
lipid oxidation
glucose metabolic process
monocarboxylic acid metabolic process
cellular_component
fatty acid metabolic process
extracellular region
extracellular region part
molecular_function
fatty acid oxidation
extracellular space
catalytic activity
oxidoreductase activity

Figure S7

Figure S7. GO term enrichment analysis of protein level differences in mouse and naked mole rat cells. Network map of GO terms enriched within proteins that have higher expression levels in naked mole rat cells compared to mouse cells. The color scaling indicates p-values of the enrichment. The figure was generated using GOrilla (2).

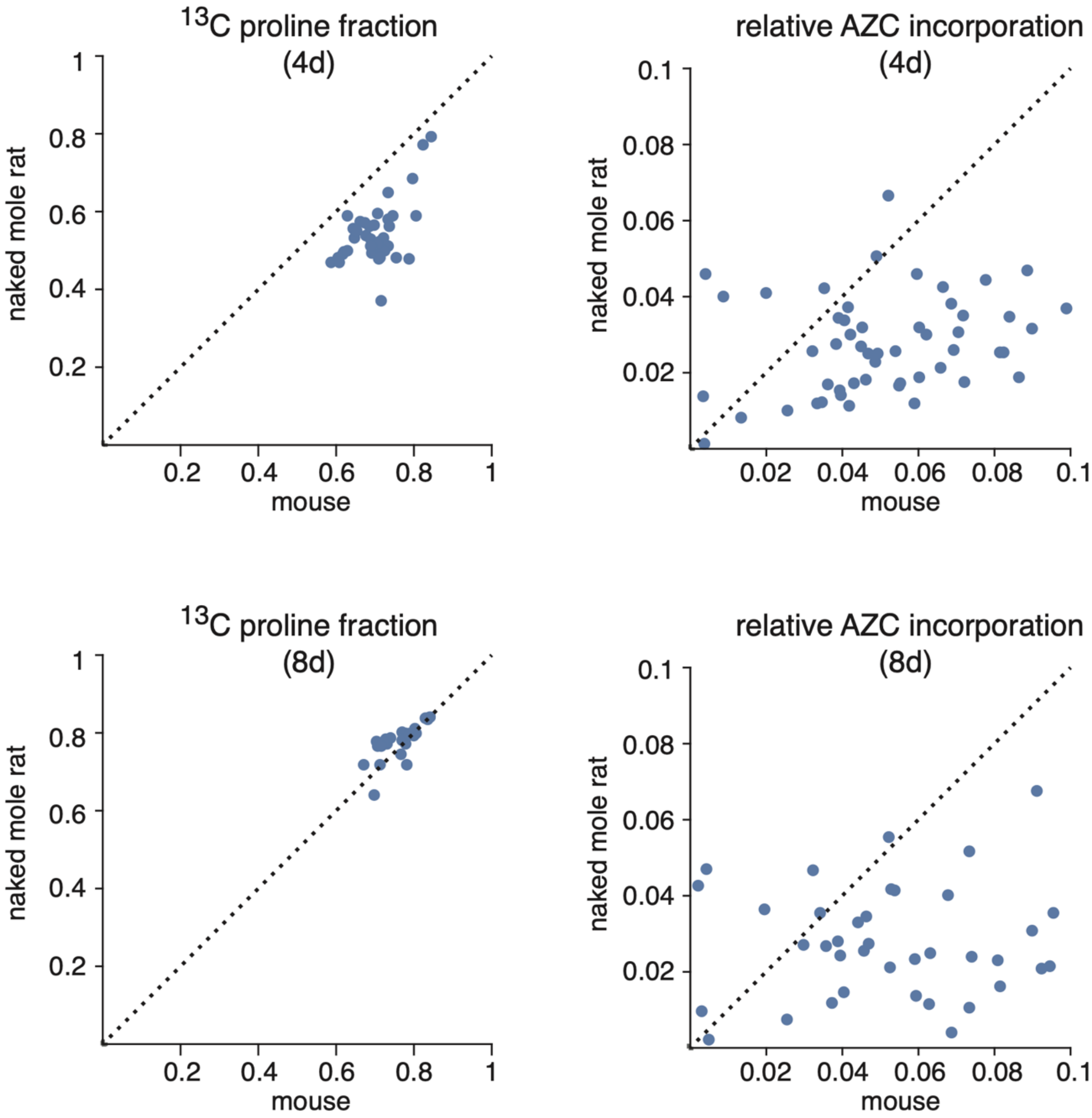

13C proline fraction (4d)
relative AZC incorporation (4d)
13C proline fraction (8d)
relative AZC incorporation (8d)
naked mole rat
mouse
0.2
0.4
0.6
0.8
1
0.02
0.04
0.06
0.08
0.1

Figure S8

Figure S8. ^{13}C proline and AZC incorporation in mouse and naked mole rat cells. Pairwise comparisons of ^{13}C proline (left) and AZC (right) incorporation for peptides quantified in both mouse and naked mole rat cells. Each point indicates a unique peptide quantified in both species on the specified day. Dotted line indicates the line of identity.

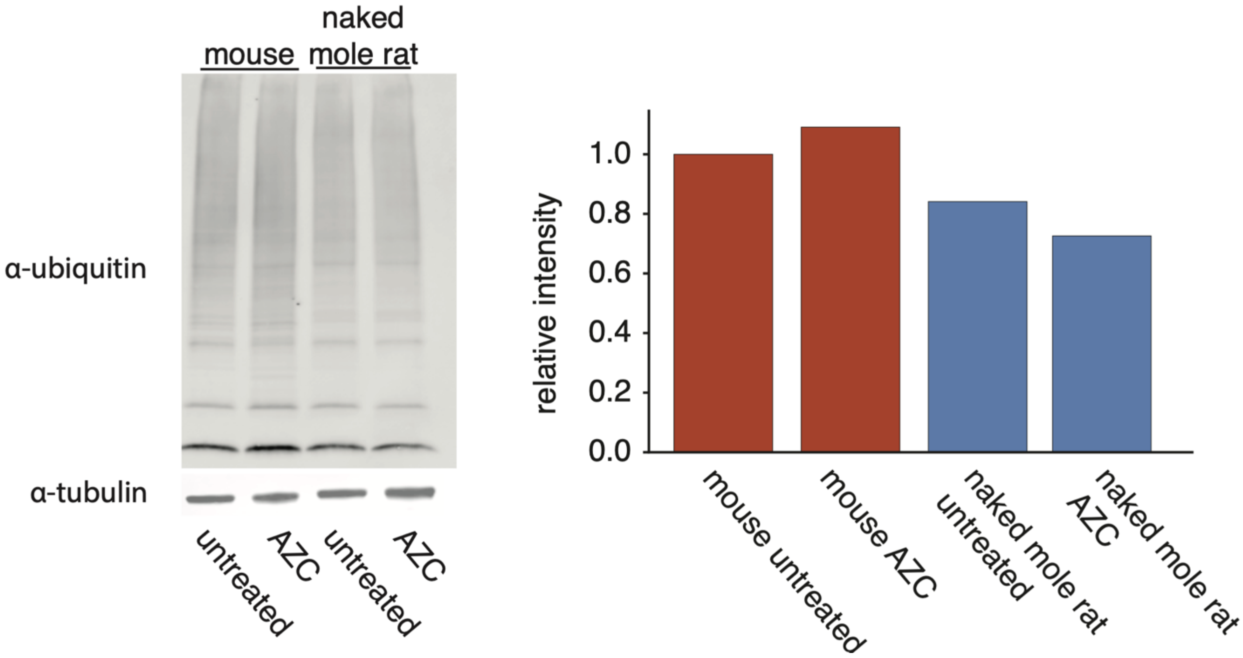
naked
mouse
mole rat
α-ubiquitin
α-tubulin
untreated
AZC
untreated
AZC
relative intensity
1.0
0.8
0.6
0.4
0.2
0.0
mouse untreated
mouse AZC
naked mole rat untreated
naked mole rat AZC

Figure S9

Figure S9. Ubiquitination in response to AZC in mouse and naked mole rat cells. Cells were treated with AZC and extracts were collected after 4 days and analyzed via western blot using an anti-ubiquitin antibody. An anti-tubulin was used as a loading control. Right indicates the quantification of the western blot. Band intensities were normalized to untreated mouse cells.

Supplementary Tables

Experiment	Time-point	Detected		H/L quantified		k_{deg} measured	
		Unique peptides	Protein groups	Unique peptides	Protein groups	Unique peptides	Protein groups
Human	2d	25881 (12370)	4766 (3116)	18523 (9585)	2951 (1780)	16091 (8018)	2671 (1586)
	4d	29539 (14008)	5031 (3275)	21149 (9934)	2934 (1745)		
	6d	26394 (13418)	4829 (3219)	17458 (8889)	2657 (1601)		
Cow	2d	34025 (16372)	4581 (3083)	26322 (13019)	3224 (2002)	16978 (8578)	2688 (1737)
	4d	30458 (15243)	4240 (2861)	23136 (11656)	2798 (1803)		
	6d	28758 (14387)	4033 (2784)	21605 (11042)	2660 (1727)		
Humpback Whale	2d	20932 (16245)	3459 (3125)	16014 (12559)	2302 (1949)	7926 (6181)	1938 (1652)
	4d	16932 (15165)	3319 (2888)	14735 (11438)	2079 (1781)		
	6d	18584 (14507)	3219 (2769)	13687 (10722)	1946 (1663)		
Bowhead Whale rep 1	2d	18136 (14027)	3489 (2885)	13733 (10686)	2218 (1818)	10194 (7795)	1756 (1547)
	4d	18324 (13882)	3421 (2912)	14022 (10675)	2150 (1779)		
	6d	16781 (12877)	3141 (2641)	12734 (9775)	2011 (1665)		
Bowhead Whale rep 2	2d	20237 (15371)	3548 (3012)	15532 (11898)	2349 (1954)	9866 (7675)	1742 (1608)
	4d	18628 (14347)	3338 (1796)	14426 (11070)	2157 (1794)		
	6d	16336 (12744)	3154 (2755)	12281 (9668)	1981 (1642)		

Table S1. Coverage of dynamic SILAC experiments. "Unique peptides" indicates the number of unique peptide sequences. "Protein groups" indicates the number of homologous protein groups. The reported number of quantified protein groups is limited to those for which heavy to light ratios (H/L) could be quantified by two independent spectra. The number of reported k_{deg} values measured for protein groups is limited to those where two distinct peptides could be measured in two or more time-points and the least squares fit to a first-order equation had an R2 > 0.80. Numbers not in parentheses correspond to searches against species-specific sequence databases, or the most closely related sequence database available. Numbers in parentheses correspond to searches conducted against the M. musculus sequence database. All subsequent analyses were conducted using the M. musculus searches in order to compare an overlapping set of peptides shared between all species. However, the results from the species-specific searches are available on the PRIDE database (accession number PXD018325).

References

1. Swovick K, Welle KA, Hryhorenko J, Seluanov A, Gorbunova V, Ghaemmaghami S. Cross-species comparison of proteome turnover kinetics. Molecular & Cellular Proteomics. 2018:mcp.RA117.000574.

2. Eden E, Navon R, Steinfeld I, Lipson D, Yakhini Z. GOrilla: a tool for discovery and visualization of enriched GO terms in ranked gene lists. BMC Bioinformatics. 2009;10:48.
